# UHRF1-mediated ferroptosis promotes pulmonary fibrosis via epigenetic repression of GPX4 and FSP1 genes

**DOI:** 10.1038/s41419-022-05515-z

**Published:** 2022-12-24

**Authors:** Yi Liu, Demin Cheng, Yue Wang, Sichuan Xi, Ting Wang, Wenqing Sun, Guanru Li, Dongyu Ma, Siyun Zhou, Ziwei Li, Chunhui Ni

**Affiliations:** 1 Gusu School, Nanjing Medical University, Nanjing, 211166 China; 2 Department of Occupational Medical and Environmental Health, Key Laboratory of Modern Toxicology of Ministry of Education, School of Public Health, Nanjing Medical University, Nanjing, 211166 China; 3 Thoracic Epigenetics Section, Thoracic Surgery Branch, National Cancer Institute, NIH, Bethesda, MD 20892 USA; 4 Department of Pathology, Nanjing Drum Tower Hospital, The Affiliated Hospital of Nanjing University Medical School, Nanjing, 210000 China

**Keywords:** Cell death, DNA methylation, Respiratory tract diseases, Predictive markers

## Abstract

Pulmonary fibrosis (PF), as an end-stage clinical phenotype of interstitial lung diseases (ILDs), is frequently initiated after alveolar injury, in which ferroptosis has been identified as a critical event aggravating the pathophysiological progression of this disease. Here in, a comprehensive analysis of two mouse models of pulmonary fibrosis developed in our lab demonstrated that lung damage-induced ferroptosis of alveolar epithelial Type2 cells (AEC2) significantly accumulates during the development of pulmonary fibrosis while ferroptosis suppressor genes GPX4 and FSP1 are dramatically inactivated. Mechanistically, upregulation of de novo methylation regulator Uhrf1 sensitively elevates CpG site methylation levels in promoters of both GPX4 and FSP1 genes and induces the epigenetic repression of both genes, subsequently leading to ferroptosis in chemically interfered AEC2 cells. Meanwhile, specific inhibition of UHRF1 highly arrests the ferroptosis formation and blocks the progression of pulmonary fibrosis in both of our research models. This study first, to our knowledge, identified the involvement of Uhrf1 in mediating the ferroptosis of chemically injured AEC2s via de novo promoter-specific methylation of both GPX4 and FSP1 genes, which consequently accelerates the process of pulmonary fibrosis. The above findings also strongly suggested Uhrf1 as a novel potential target in the treatment of pulmonary fibrosis.

## Introduction

As an end-stage clinical phenotype of interstitial lung diseases (ILDs), pulmonary fibrosis (PF) is characterized by loss of alveolar gas-exchange function and excessive deposition of the extracellular matrix (ECM) [1]. During the development of PF, persistent inflammation at the injured site of the alveoli is accompanied by the accumulation of activated pulmonary fibroblasts. The abnormal repair of alveoli leads to the progressive loss of respiratory function and even death [2]. Due to the high economic burden and frequent common complications of lung transplantations, patients with PF are rarely recovered in the clinics. Also, there are few clinical therapeutic drugs available currently [3, 4].

Pulmonary fibrosis could be resulted from both identified and unidentified causing factors. The most serious PF form with unknown cause is idiopathic pulmonary fibrosis (IPF), which manifests with the worst prognosis with a median survival of 2–5 years after diagnosis [5]. The prevalence and associated mortality of IPF, usually occurring in the elderly, is rising world widely [1]. The currently identified inducers of PF include occupational exposure and viruses, such as silica exposure-caused silicosis [6, 7]. Despite the heterogeneity and variability in the clinical diagnosis and prognosis of different types of pulmonary fibrosis, common signal pathways and pathological processes are shared [2, 8].

Alveolar epithelium cells injured in IPF or other types of PF are considered as an initial event of fibrogenesis [9]. Since alveolar epithelial type 2 cell (AEC2) could proliferate and differentiate into alveolar epithelial type 1 cells (AEC1) during normal alveoli repair, the loss of AEC2 is recognized as a key factor of PF [10]. Former studies suggested AEC2 death, either apoptotic, necrotic [10] or ferroptotic [11], contributes to PF pathogenesis. However, the molecular signals regulating ferroptosis in AEC2 and their surrounding cells remain elusive in PF. Ferroptosis, as a relatively new form of programmed cell death, is distinct from other forms of cell death in morphology, genetics, and biochemistry [12]. Ferroptosis applies an overwhelming lipid peroxidation to induce iron-dependent cell death, which plays diverse roles in the pathophysiological processes of multiple lung diseases, including lung cancer, chronic obstructive pulmonary disease (COPD), and lung fibrosis [13].

To comprehensively investigate the roles of AEC2 ferroptosis in PF, we examined the pro-PF function of AEC2 ferroptosis in silica- and bleomycin-induced mouse PF models as well as silica-treated primary mouse AEC2s and A549 cells. We found that both GPX4 and FSP1 regulated AEC2 ferroptosis in a promoter DNA methylation-dependent manner, and our transcriptome sequencing analysis further identified UHRF1 as a key molecule in ferroptosis-related DNA methylation. Furthermore, we demonstrated the application of liposomal Uhrf1 siRNA to intervene mouse PF with high transfection efficiency and a long-lasting silencing effect. The results from PF patient’s lung tissue and primary AEC2 organoid culture further confirmed our findings. Together, our data reveal the critical roles of AEC2 ferroptosis in PF and delineate the potential molecular mechanism for developing UHRF1 as a potential therapeutic target.

## Results

### Ferroptosis is involved in pulmonary fibrosis in vivo

**Fig. 1:**
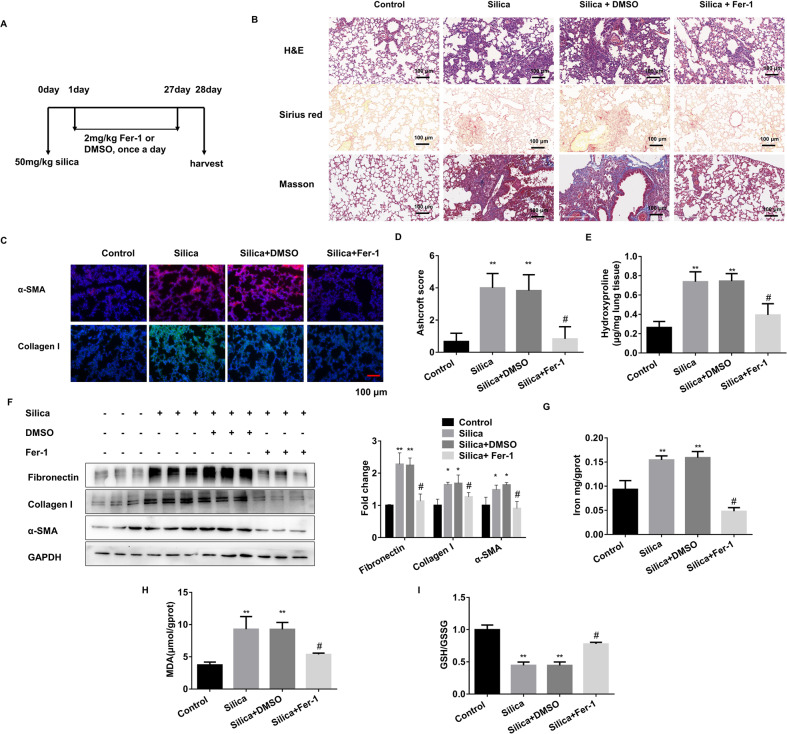
Ferroptosis inhibitor Fer-1 alleviates silica-induced PF in vivo. **A** Schematic diagram of Fer-1 or DMSO-treated mouse silicosis model. Mice from control, 50 mg/kg silica, 50 mg/kg silica with 2 mg/kg Fer-1, and 50 mg/kg silica with DMSO-treated groups were sacrificed on day 28. **B** Representative hematoxylin/eosin (HE), Sirius red, and Masson staining of lung tissues from each group were presented (scale bars, 100 µm). **C** Representative immunofluorescence staining of α-SMA and Collagen I from mouse lung tissues (scale bars, 100 µm). **D** Ashcroft score of mice from each group. **E** The levels of hydroxyproline content were determined at 550 nm and expressed as micrograms per mg of lung tissues, determined by the hydroxyproline content assay kit. **F** The protein levels of Fibronectin, Collagen I, and α-SMA in lung tissues from each group were detected by western blot and qualified (means ± SD, *n* = 3). **G** The labile iron concentration of lung tissues was assessed using an Iron Colorimetric Assay Kit. **H** MDA concentration lung tissues was measured using a Lipid Peroxidation MDA Assay Kit. **I** GSH/GSSG ratio of lung tissues. *P < 0.05, **P < 0.01 versus control group, ^#^*P* < 0.01 versus silica+DMSO treated group.

Based on the successful establishment of two pulmonary fibrosis models via intra-tracheal instillation of silica and bleomycin (BLM), we further established the intervention mouse models by using ferroptosis inhibitor Ferrostatin-1 (Fer-1). Firstly, we demonstrated that Fer-1 or DMSO administration showed no significant toxic effect in C57BL/6 mice (Supplementary Fig. 1). Then, the Fer-1 intervention was performed in both silica or BLM-induced PF models (Fig. 1A, Supplementary Fig. 2A). HE staining revealed pulmonary fibrosis in both models, while the fibrotic areas were different. BLM-induced PF areas were closer to the lung edge (Supplementary Fig. 2B), whereas the silica-induced PF areas were located around the bronchi (Fig. 1B). This phenomenon is consistent with previous research findings [6, 14]. Meanwhile, both α-SMA staining and Ashcroft score analysis showed that Fer-1 alleviated the aggravation of PF (Fig. 1C, D, Supplementary Fig. 2C). Sirius red staining and Masson staining assays further confirmed that the changes in collagen deposition were consistent with the PF degree (Fig. 1B, Supplementary Fig. 2B). In addition, fibrosis indicators and hydroxyproline content were upregulated in the lung tissues of PF models significantly reduced with exposure to Fer-1 treatment (Fig. 1E, F, Supplementary 2D, E). The iron accumulation was accelerated in the fibrotic groups and dramatically arrested in the Fer-1 groups (Fig. 1G, Supplementary Fig. 2F). Further, MDA and GSH/GSSH levels were used individually to detect the lipid peroxide products and antioxidant activity in lung tissues (Fig. 1H&I, Supplementary Fig. 2G, H). Together, the Fer-1 interrupted groups showed anti-lung fibrosis phenotype in the PF models. Meanwhile, the total Gpx4 and Fsp1 mRNA levels in the lung tissues were almost unchanged in different groups (Supplementary Fig. 2I).

### In vitro chemical exposure induces AEC2s undergoing ferroptosis

**Fig. 2:**
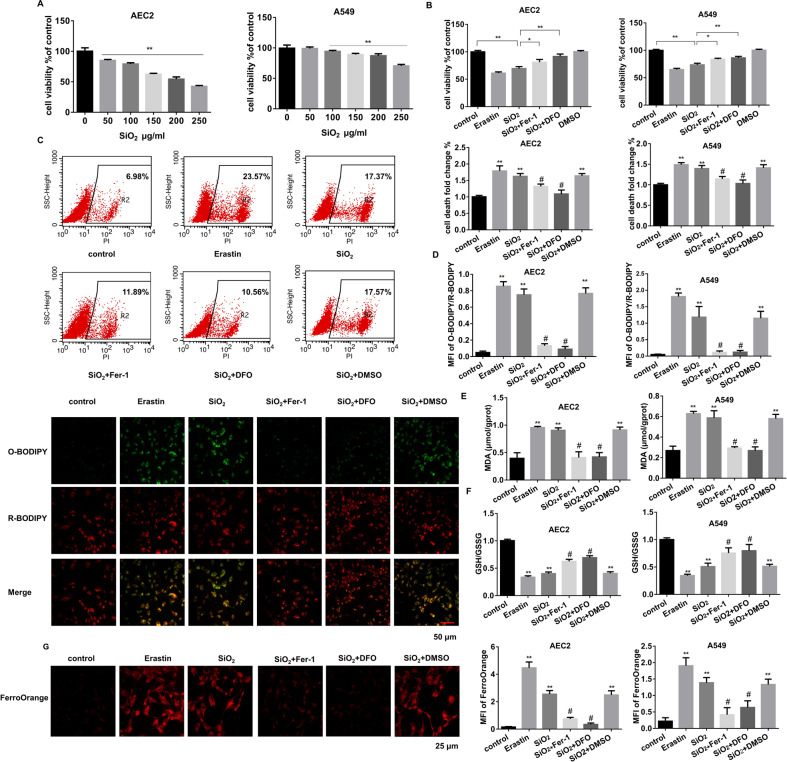
AEC2s undergo ferroptosis upon SiO_2_ stimulation. **A** CCK8 detected mouse primary AEC2 and A549 cell viability after treated with 0, 50, 100, 150, 200, and 250 μg/mL SiO_2_ for 24 h (**P < 0.01). Then AEC2 and A549 cells were treated with 1 μM erastin, 150 or 250 µg/mL SiO_2_ individually, SiO_2_ with 1 μM Fer-1, SiO_2_ with 100 μM DFO, and DMSO for 24 h. CCK8 (**B**), flow cytometry representative pictures of primary AEC2 and quantification results of both primary AEC2 and A549 cells (**C**) were shown. **D** Representative images of C11-BODIPY in treated-primary AEC cells (*red*: reduced C11-BODIPY, *green*: oxidized C11-BODIPY; scale bars, 50 µm; lower panel), and quantification of C11-BODIPY fluorescence in primary AEC2 and A549 cells (upper panel). **E** MDA concentration in cell lysates from primary AEC2 and A549 cells was measured using a Lipid Peroxidation MDA Assay Kit. **F** GSH/GSSG ratio of primary AEC2 and A549 cells. **G** Intracellular Fe^2+^ was detected with the FerroOrange probe, and representative images of primary AEC2 cells were shown (*red*: FerroOrange-stained Fe^2+^; scale bars, 25 µm; left panel). Fe^2+^ fluorescence intensity was quantified by ImageJ in both primary AEC2 and A549 cells (right panel). *P < 0.05, **P < 0.01 versus control group, ^#^*P* < 0.01 versus SiO_2_ + DMSO treated group.

To determine which cells undergo ferroptosis during PF, we isolated primary mouse AEC2s and lung fibroblast cells. Silica was used to treat the THP-1-induced macrophages and AEC2s, while primary fibroblast cells were exposed to TGF-β1 (Supplementary Fig. 3B). Since AEC2 cells tend to differentiate into AEC1 cells in vitro, the A549 cell line was also used along with primary mouse AEC2 cells considering its AEC2 character. Cell viability assay demonstrated an increased cell death rate of both AEC2s and macrophages after treatment (Fig. 2A, Supplementary Fig. 3C), while fibroblast proliferation increased (Supplementary Fig. 3C). Then the inhibitors of apoptosis, ferroptosis, and necrosis were applied to further define the type of cell death. 1 μM Erastin was used to induce ferroptosis in cells, while 1 μM Fer-1 or 100 μM DFO was used to reverse the fibrotic phenomenon. Their IC50 values were shown in Supplementary Fig. 3A. It was found that macrophages mainly underwent apoptosis, and ferroptosis occurred only in AEC2s (Fig. 2B, Supplementary Fig. 3D). Meanwhile, apoptosis may also contribute to silica-induced AEC2 cell death since its inhibitor also partly reversed the outcome. Flow cytometry analysis showed that erastin-induced ferroptosis aggravated cell death in both primary AEC2s and A549, which is similar to the SiO_2_ treatment. In the meantime, its inhibitor Fer-1 or DFO blocked SiO_2_-stimulated ferroptosis (Fig. 2C). Elevation of lipid ROS generation (C11-BODIPY assay), lipid peroxidation product MDA, and labile iron pool (LIP, FerroOrange assay) as well as GSH/GSSH ratio were observed under SiO_2_ or erastin stimulation in primary AEC2s and A549 cells (Fig. 2D–G, Supplementary Fig. 3E, F), in which all those were reversed by ferroptosis inhibitor Fer-1. These results suggest that ferroptosis may play its role in AEC2s during the development of PF.

We further examined whether GPX4 and FSP1 play critical roles in regulating the ferroptosis of macrophages and fibroblasts. SiO_2_ treatments at different concentrations didn’t alter GPX4 and FSP1 mRNA levels in macrophages (Supplementary Fig. 4B). Interestingly, GPX4 levels were also not significantly changed in primary fibroblasts and MRC5 fibroblast cell lines, but FSP1 showed an upward trend after being treated with TGF-β1 (Supplementary Fig. 4C). To further explore its function in fibroblasts with siRNA-mediated depletion of FSP1 (Supplementary Fig. 4D, E), we found that FSP1 downregulation decreased cell viability in TGF-β1 or erastin-treated fibroblasts, which can be recovered by the ferroptosis inhibitor Fer-1 to some extent (Supplementary Fig. 4F, G). The above results reveal the protective role of FSP1 on ferroptosis in lung fibroblasts.

### Deregulated GPX4 and FSP1 contribute to SiO_2_-induced AEC2 ferroptosis

**Fig. 3:**
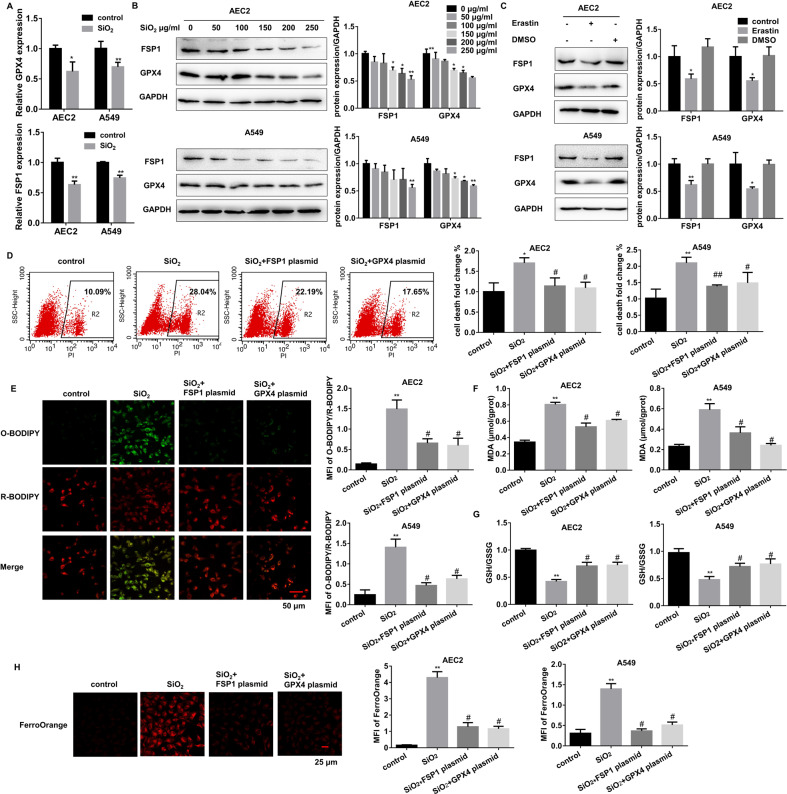
GPX4 cooperates with FSP1 to regulate SiO_2_-induced AEC2 ferroptosis. **A** mRNA levels of GPX4 and FSP1 were measured in 150 µg/mL SiO_2_ treated primary AEC and 250 µg/mL SiO_2_ treated A549 cells (*P < 0.05 versus control group, **P < 0.01 versus control group). **B** Representative immunoblotting of GPX4 and FSP1 in 0, 50, 100, 150, 200, and 250 µg/mL SiO_2_ treated primary AEC_2_ and A549 cells (left panel), means ± SEM of three independent experiments (right) are shown. **C** Mouse primary AEC2 and A549 cells were treated with 1 μM erastin or DMSO, representative immunoblotting of GPX4 and FSP1 (left), as well as means ± SEM of three independent experiments (right) are shown. Then AEC2 and A549 cells were treated with 150 or 250 µg/mL SiO_2_ individually, SiO_2_ with FSP1 plasmid, and SiO_2_ with GPX4 plasmid. **D** Flow cytometry representative pictures of primary AEC2 and quantification results of both primary AEC2 and A549 cells. **E** Representative images of C11-BODIPY in treated-primary AEC cells (*red*: reduced C11-BODIPY, green: oxidized C11-BODIPY; scale bars, 50 µm; left panel), and quantification of C11-BODIPY fluorescence in primary AEC2 and A549 cells (right panel). **F** MDA concentration in cell lysates from primary AEC2 and A549 cells was measured using a Lipid Peroxidation MDA Assay Kit. **G** GSH/GSSG ratio of primary AEC2 and A549 cells. **H** Intracellular Fe^2+^ was detected with the FerroOrange probe, and representative images of primary AEC2 cells were shown (*red*: FerroOrange-stained Fe^2+^; scale bars, 25 µm; left panel). Fe^2+^ fluorescence intensity was quantified by ImageJ in both primary AEC2 and A549 cells (right panel). **P < 0.01 versus control group, ^#^*P* < 0.01 versus SiO_2_-treated group.

To explore the contribution of deregulated GPX4 and FSP1 to SiO_2_-induced AEC2 ferroptosis, we examined AEC2s treated with 0~250 μg/mL SiO_2_ for 24 h and found that the GPX4 and FSP1 mRNA and protein levels were reduced in a dose-dependent manner (Fig. 3A, B). Similarly, erastin also decreased both GPX4 and FSP1 levels (Fig. 3C). We also found a slightly increasing trend of SLC7A11 in SiO_2_-treated primary AEC2s (Supplementary Fig. 4A), which might be caused by the compensatory effect. Then we used over-expression plasmids to restore GPX4 and FSP1 expression in SiO_2_-stimulated AEC2s. Both RNA and protein levels showed successful recovery (Supplementary Fig. 4I–J). The restoration of either GPX4 or FSP1 could dramatically and respectively decrease SiO_2_-induced AEC2 ferroptosis, as shown by the results of cell flow cytometry-detected cell death (Fig. 3D), Lipid ROS generation (Fig. 3E), MDA (Fig. 3F), GSH depletion (Fig. 3G), and LIP accumulation (Fig. 3H). These data collectively demonstrate that deregulated GPX4 or FSP1 contributes to external stimuli-caused damage via mediating ferroptosis in AEC2s.

### De novo promoter methylation mediates the repression of both GPX4 and FSP1 leading to ferroptosis of AEC2s

**Fig. 4:**
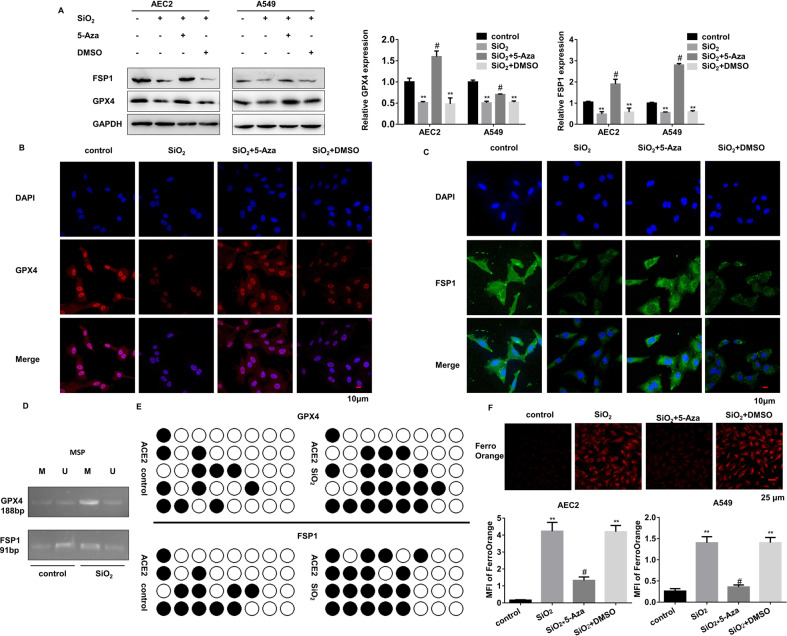
DNA-methylation occurs in GPX4 and FSP1 promoter regions in SiO_2_-stimulated AEC2s. Primary AEC2 and A549 cells were treated with SiO_2_, SiO_2_ + 5-Aza, and SiO_2_ + DMSO. **A** Protein levels of GPX4 and FSP1 were detected by western blot and qualified (means ± SD, *n* = 3) (**P < 0.01 versus control group, ^#^*P* < 0.01 versus SiO_2_ + DMSO group). **B** Representative immunofluorescence staining of GPX4 (red) and **C** FSP1 (green) in primary AEC2 cells were shown, scale bars, 10 μm. **D** Methylation-specific PCR detected DNA methylation levels of ACE2 cells with or without SiO_2_. **E** DNA from control and SiO_2_-treated AEC2 cells were subjected to bisulfite sequencing of 5′UTR. Methylated CpG sites are shown with black circles and unmethylated sites with open circles. **F** Intracellular Fe^2+^ was detected with the FerroOrange probe, and representative images of primary AEC2 cells were shown (red: FerroOrange-stained Fe^2+^; scale bars, 25 µm; upper panel). Fe^2+^ fluorescence intensity was quantified by ImageJ in both primary AEC2 and A549 cells (**P < 0.01 versus control group, ^#^*P* < 0.01 versus SiO_2_ + DMSO group; lower panel).

To figure out the upstream regulatory mechanisms of GPX4 and FSP1, we first detected their genomic DNA levels by using primary mouse AEC2s derived from the in-vivo PF models and recognized no changes in DNA copy number among these mice (Supplementary Fig. 5A). Then we tested whether epigenetic regulations of GPX4 and FSP1are involved in their transcriptional activities. The online prediction screening found that there are relatively abundant methylation sites in their gene promoter regions (Supplementary Fig. 5B). DNA methylation inhibitor successfully recovered GPX4 and FSP1 protein levels in silica-treated AEC2s (Fig. 4A–C, Supplementary Fig. 5C, D). The promoter-specific MSP and BSP further proved higher DNA methylation levels in stimulated primary AEC2s (Fig. 4D, E). Further DNA methylation intervention in primary AEC2s also dismissed the ferroptosis incidence, as suggested by LIP (Fig. 4F). Thus, we propose that DNA methylation levels of GPX4 and FSP1 are elevated in stimulated AEC2s, which induces ferroptosis.

### Identification of UHRF1 as a specific methylation regulator for silencing GPX4 and FSP1 genes

**Fig. 5:**
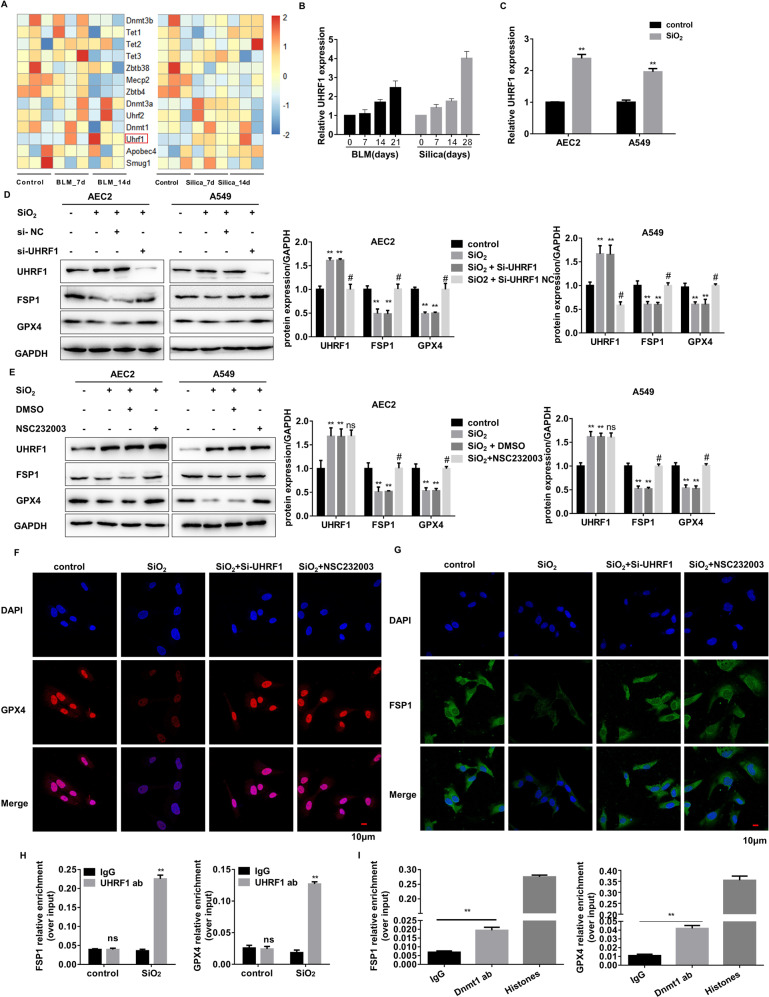
UHRF1 exerts DNA methylation recruitment function in GPX4 and FSP1 genes. **A** A heat map representing the differentially expressed DNA methylation related-RNA in the bleomycin or silica-treated mice lung tissues. The up-regulated mRNAs are indicated in progressively brighter shades of red, and the down-regulated mRNAs are indicated in progressively brighter shades of blue (*n* = 3). **B** qRT-PCR of Uhrf1 RNA levels in bleomycin-treated 0, 7, 14, and 21 days mouse lung tissues, and silica-treated 0, 7, 14, and 28 days mouse lung tissues (**P < 0.01 versus control group). **C** qRT-PCR of Uhrf1 RNA levels in 150 µg/mL SiO_2_-treated primary AEC2 cells or 250 µg/mL SiO_2_-treated A549 cells (**P < 0.01 versus control group). **D** Protein levels of UHRF1, GPX4, and FSP1 were detected by western blot and qualified (means ± SD, *n* = 3) after being treated with SiO_2_, SiO_2_ + UHRF1 siRNA, or SiO_2_ + NC siRNA (**P < 0.01 versus control group, ^#^*P* < 0.01 versus SiO_2_ + NC siRNA group). **E** Protein levels of UHRF1, GPX4, and FSP1 were detected by western blot and qualified (means ± SD, n = 3) after treated with SiO_2_, SiO_2_ + NSC232003, or SiO_2_ + DMSO (**P < 0.01 versus control group, ^#^*P* < 0.01 versus SiO_2_ + DMSO group). **F** Representative immunofluorescence staining of GPX4 (red) and **G** FSP1 (green) in SiO_2_, SiO_2_ + UHRF1 siRNA, or SIO_2_ + NSC232003-treated primary AEC2 cells were shown, scale bars, 10 μm. ChIP assay detected UHRF1 (**H**) enrichment of the promoter region of GPX4/FSP1 in control and SiO_2_-treated AEC2 cells, and DNMT1 (**I**) enrichment of the promoter region of GPX4/FSP1 in SiO_2_-treated AEC2 cells (**P < 0.01 versus IgG group).

RNA-sequencing was performed to examine potential DNA methylation regulators for silencing GPX4 and FSP1. Briefly, lung tissue RNA was extracted from the mice treated with silica or bleomycin at 0, 7, 14 days. DNA methylation-related regulatory genes were listed in the heatmap (Fig. 5A). Most genes didn’t show a significant or consistent change in the mRNA level. However, the expression of UHRF1 increased with the development of PF (Fig. 5B). In-vitro silica treatment also elevated UHRF1 levels in AEC2s (Fig. 5C). Therefore, we speculated that UHRF1 might be involved in regulating the DNA methylation in AEC2s. Its downstream genes were first examined using UHRF1 siRNA (Supplementary Fig. 6A). Knockdown of UHRF1 did restore SiO_2_-induced reductions in GPX4 and FSP1 (Fig. 5D–G, Supplementary Fig. 6G, H), acting similarly to UHRF1 inhibitor, which inhibits DNA methylation via disrupting DNMT1/UHRF1 interactions in vitro. However, UHRF1 blockage didn’t affect other ferroptosis-relator genes, including Nrf2, Ncoa4, Hspb1, Scl7a11, and Trf1 (Supplementary Fig. 6B–F). Also, UHRF1 downregulation reversed ferroptosis in stimulated AEC2s (Supplementary Fig. 6I–K). CHIP experiments were performed to investigate whether UHRF1 directly binds and regulates the genes through DNA methylation. The result demonstrated the significant enrichment of UHRF1 in Gpx4 and Fsp1 promoter regions in silica-stimulated AEC2s (Fig. 5H). Meanwhile, methyltransferase DNMT1 was also recruited to the promoter regions with UHRF1 in SiO_2_-treated AEC2. (Fig. 5I). Taken together, UHRF1 promotes ferroptosis by recruiting DNMT1 to Gpx4 and Fsp1 genes, thereby leading to de novo DNA methylation in their promoter regions when AEC2s cells are stimulated.

### UHRF1 targeted liposomal siRNA balks PF progression in vivo

**Fig. 6:**
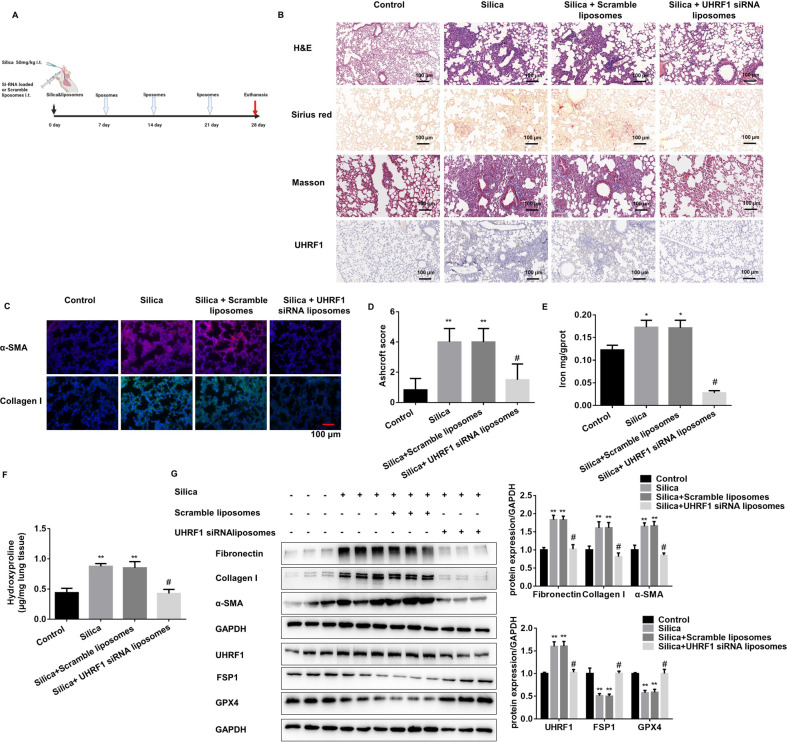
UHRF1 liposomal siRNA could block PF progression in mouse models. **A** Schematic diagram of UHRF1 siRNA liposome or scramble liposome together with silica-treated mouse experiment. **B** Representative HE, Sirius red, UHRF1 IHC, and Masson staining of lung tissues from each group of C57BL/6 mice sacrificed on day 28 (scale bars, 100 µm). **C** Representative immunofluorescence staining of α-SMA and Collagen I from mouse lung tissues (scale bars, 100 µm). **D** Ashcroft score of mice from each group. **E** The labile iron concentration of lung tissues was assessed using an Iron Colorimetric Assay Kit. **F** The levels of hydroxyproline content were determined at 550 nm and expressed as micrograms per mg of lung tissues, determined by the hydroxyproline content assay kit. **G** The protein levels of Fibronectin, Collagen I, α-SMA in lung tissues, and UHRF1, FSP1, GPX4 in primary AEC2 cells from each group were detected by western blot and qualified (means ± SD, *n* = 3). *P < 0.05 versus control group, **P < 0.01 versus control group, ^#^*P* < 0.01 versus silica+scramble liposomes group.

Given that cationic liposome is an ideal carrier for the treatment of PF, Uhrf1 siRNA-loaded liposome was prepared for the in-vivo study. The Uhrf1 siRNA liposomes were characterized by a diameter of around 164 nm with an 80.4% entrapment efficiency (Supplementary Fig. 7A, B). Moreover, the liposomes exhibited a round and uniform bilayer membrane structure under TEM (Supplementary Fig. 7C). To better track its location in vivo, liposomes were labeled with Dir fluorescence (Supplementary Fig. 7D). And its stability could be maintained in vivo for at least 7 days (Supplementary Fig. 7E). In vitro validation experiments showed that the liposomes could enter the primary AEC2s without cytotoxicity (Supplementary Fig. 7F, G). Also, UHRF1 siRNA liposomes administration showed no in vivo toxicity (Supplementary Fig. 7K–O). Next, the Uhrf1 siRNA liposomes were injected intratracheally every 7 days after SiO_2_ or BLM treatment in mice (Fig. 6A, Supplementary Fig. 7H). After 28 days of silica or 21 days of BLM treatment, the mice were sacrificed. Significant liposome fluorescence could be found in the lung area of UHRF1 siRNA-treated mice, and mainly concentrated in the lung area (Supplementary Fig. 7I, J). The results indicated that UHRF1 intervention reduced PF degree in both mouse models (Fig. 6B–F, Supplementary Fig. 8A–D), and also decreased ferroptotic indicators (Supplementary Fig. 8E–G). At the same time, the GPX4 and FSP1 expression was derepressed with the UHRF1 siRNA liposome treatment in primary ACE2 cells (Fig. 6G, Supplementary Fig. 8H). The results of our in vivo experiments suggest that UHRF1 siRNA liposomes may be a promising potent therapeutic option for PF.

### The pivotal role of UHRF1 in 3D model and clinical samples

**Fig. 7:**
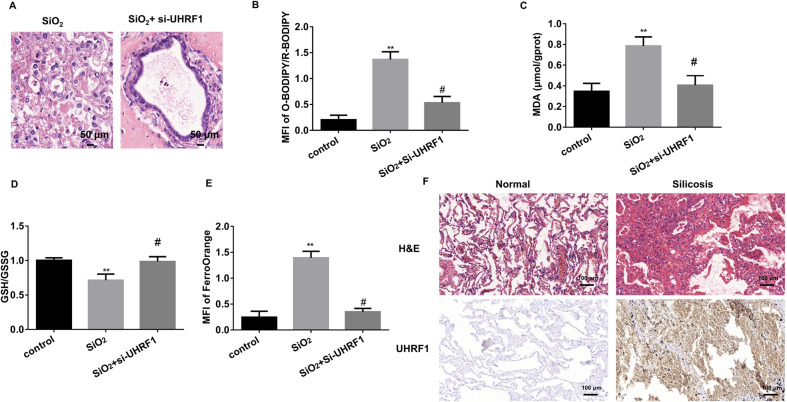
AEC2 3D model confirms UHRF1-regulated ferroptosis in PF. **A** Representative HE staining of primary AEC2 cell 3D culture treated by SiO_2_ with or without UHRF1 siRNA for 14 days. **B** quantification of C11-BODIPY fluorescence in primary AEC2 organoid cells (**P < 0.01 versus control group, ^#^*P* < 0.01 versus SiO_2_-treated group). **C** MDA concentration in cell lysates from primary AEC2 organoid (**P < 0.01 versus control group, ^#^*P* < 0.01 versus SiO_2_-treated group). **D** GSH/GSSG ratio of primary AEC2 organoid cells (**P < 0.01 versus control group, ^#^*P* < 0.01 versus SiO_2_-treated group). **E** Intracellular Fe^2+^ fluorescence intensity was quantified in both primary AEC2 organoid cells (**P < 0.01 versus control group, ^#^*P* < 0.01 versus SiO_2_-treated group; right panel). **F** Representative HE and UHRF1 IHC staining in normal and silicosis patient lung tissues (scale bars, 100 µm).

To overcome the limitations of 2D culture, we also performed 3D culture by using primary mouse AEC2 and fibroblast cells. HE staining showed that silica treatment disrupted the organoid formation of AEC2s, while UHRF1 knockdown could restore its organoid-formation ability (Fig. 7A). Depletion of UHRF1 inhibited SiO_2_-induced ferroptosis markers in the AEC2 organoid (Fig. 7B–E). Also, HE and UHRF1 IHC staining were performed in the collected silicosis patient lung samples. Immunostaining intensity of UHRF1 was found to be broadly expressed in silicosis patient lungs, especially in the fibrotic area (Fig. 7F), further implying its vital role in PF.

## Discussion

As a newly identified form of programmed cell death, ferroptosis is involved in the process of various diseases. Research mainly focused on its potential treatment role by targeting the antioxidant capacity of tumor cells, which leads to tumor cell death [15]. Recently, it has been reported that ferroptosis also participates in tumor immunity [16]. Given that the damaged alveolar structure is an important pathogenic factor in both IPF and occupational PF [17], the involvement of ferroptosis in those diseases was investigated in this study by using silica-induced silicosis-like and bleomycin-induced IPF-like mouse models. Since the pathogenesis of PF is usually characterized by immune cell-related pulmonary inflammation and activated lung fibroblast-related abnormal alveolar repair, we identified the ferroptosis specifically occurring in AEC2s rather than macrophages or fibroblasts. Our data indicate that AEC2 ferroptosis may be a convergent process in different types of PF.

Multiple genes, including GPX4, FSP1, p53, and Nrf2 (nuclear factor erythroid 2-related factor 2), regulate cell ferroptosis [15]. The canonical glutathione-dependent GPX4 pathway protects the cell from ferroptosis against membrane lipid peroxidation [18]. GPX4 could reduce hydroperoxides via its catalytic function, and the oxidized form of glutathione produced in this process is recovered by glutathione reductase and NADPH/H+. Moreover, CoQ depletion could increase lipid peroxidation and cell death upon ferroptosis-inducing agents treatment [19]. However, GPX4 inhibition fails to trigger ferroptosis in some types of tumor cells. Further studies figured out that FSP1 acts as an alternative ferroptosis suppressor in parallel to GPX4 [20, 21]. FSP1 mediates NADH-dependent reduction of CoQ on the plasma membrane, which inhibits phospholipid peroxidation. By interfering with their expression in vitro, we demonstrated that GPX4 and FSP1 jointly regulate ferroptosis in AEC2s and act as critical ferroptosis modulators in the PF progress.

Notably, the expression of GPX4 and FSP1 was relatively consistent in our mouse lung tissues and other PF patients’ lung tissues from the online database. Through the analysis of the existing data, we believe that this is caused by multiple factors. Firstly, the in vitro results suggested both GPX4 and FSP1 expression decreased in only AEC2s, not in activated macrophages or fibroblasts. The FSP1 levels were even higher in TGF-β1-treated fibroblasts. Moreover, the lung is a complex organ comprising many cell types, and the proportion of AEC2s is relatively low in the lung, especially in the fibrotic lung. These factors help explain that there are no significant changes in gross levels of GPX4 and FSP1 in PF lungs. Some studies suggested that TGF-β1 could induce fibroblast ferroptosis, which performed a fibroblast-to-myofibroblast transition (FMT)-promoting role in lung fibrosis [22, 23]. We also observed a slight decrease of GPX4 expression in activated fibroblasts, however, no ferroptosis was observed in fibroblasts here. Meanwhile, the FSP1 was elevated in TGF-β1 treated primary lung fibroblasts. Hence, it’s considered that FSP1 may protect cells from ferroptosis in the process of FMT. Knockdown of FSP1 could restore the cell death-promoting ability of eratin, which further supports our hypothesis and implicates it as a potential therapeutic target in PF.

We further studied the upstream regulation mechanism of GPX4 and FSP1. Since the gDNA levels were not affected, the alteration in their expression should be carried out at the transcriptional level. It’s identified that there are abundant CpG islands in the promoter regions of GPX4 and FSP1, and DNA-methylation is widely involved in PF progress [24]. We hypothesized that their expression may be regulated by DNA methylation. The methylation primers for promoter regions were designed. DNA methylation inhibitor was used to restore GPX4 and FSP1 in SiO_2_-treated AEC2s. Moreover, promoter-specific MSP and BSP proved higher DNA methylation levels in their promoter regions after stimulation. However, DNA methylation is regulated by multiple genes at different stages [25], we screened the potential regulator by using RNA-seq. The RNA-seq was accomplished by mouse lung tissues that were treated with silica or bleomycin for different stages. Interestingly, the levels of traditional DNA methyltransferases were not significantly changes, including DNMTs. Strikingly, the DNMT1 recruiter UHRF1 is elevated in both PF mouse models.

Recent studies have found that UHRF1 exerts its differential function through 5 domains [26]. The domain related to DNA methylation is the SRA domain, which is involved in recognizing and interacting with DNA methyltransferases (DNMT1) [27]. It’s well known that UHRF1 is tightly regulated in a cell cycle-specific manner, and consistently over-expressed in a variety of tumors [28, 29], but its role in fibrotic diseases has not been studied yet. Here it was found that UHRF1 promoted AEC2 ferroptosis by regulating the DNA methylation of GPX4 and FSP1. Inhibition of UHRF1 could restore the functions of GPX4 and FSP1. To further verify the pro-fibrotic role of UHRF1 in vivo, we constructed the liposomes that enclosed UHRF1 siRNA. Since liposome is the only inhalation nanomedicine approved by the FDA [30], liposome could deliver siRNA to lung. The advantages of liposome siRNA are obvious, it could protect siRNA from degradation with high transfection efficiency and with barely any known cytotoxicity in vivo [31]. Our UHRF1 liposome siRNA was tagged with Dir fluorescence to facilitate in vivo tracking. It showed no cytotoxicity and could maintain high levels in mouse lungs for at least 7 days. Since the tracheal perfusion method was used to deliver the liposomes, the UHRF1 liposome siRNA mainly accumulated in the lung. By injecting the liposomes every 7 days, the lung UHRF1 levels remained repressed, and PF degree was alleviated in both models.

In addition, the AEC2 organoid was constructed to simulate the alveolar structure. It turns out that UHRF1 knockdown could facilitate the formation of organoids and reduce ferroptosis. Moreover, the UHRF1 expression in the PF patients was consistent with the experimental results. At last, we also preliminarily confirmed that the expression of UHRF1 was also significantly increased in activated fibroblasts during pulmonary fibrosis. Based on this, our group will further study the mechanism of UHRF1 in FMT, and relevant experiments have been carried out. Also, pharmacokinetic experiments should be performed to detect Fer-1 or siRNA liposome concentration in lung to avoid the interference from secondary mechanisms in our further study.

Overall, the above data demonstrated that GPX4 and FSP1 act jointly to regulate the ferroptosis of AEC2 cells in PF and are regulated by UHRF1-monitored DNA methylation. Liposome UHRF1 siRNA could perform an ideal therapeutic efficiency of pulmonary fibrosis in vivo. We believe this study provides significant insight into the pathogenesis of PF and confers promising therapeutic targets and methods for its treatment.

## Materials and methods

### Ethics statement

All animal experiments were approved by Nanjing Medical University Ethics Committee (Nanjing, China), complied with the National Institutes of Health guide for the care and use of Laboratory animals (NIH Publications No. 8023). Six-week-old male C57BL/6 mice were purchased from the Animal Center of Nanjing Medical University and housed in specific pathogen-free (SPF) conditions. The mice were marked with numbers. Then excel software was used to divide the mice into groups randomly. Five Silicosis and two control patient lung tissue samples were obtained after patient consent at the Nanjing Medical University (Nanjing, China) in accordance with institutional review board protocols. The research was conducted according to the principles of the World Medical Association Declaration of Helsinki.

### Preparation and characterization of UHRF1 siRNA-loaded liposomes

UHRF1 siRNA was encapsulated by using liposomes. The lipid mixture was composed of C12-200, cholesterol, DSPC, and mPEG-DMG, which was dissolved at a molar ratio of 50:38.5:10:1.5 in ethanol. Then unentrapped siRNA was depleted by ultrafiltration centrifugation. 1 mg/kg of DiRlabelled liposomes was injected intratracheally in mice to detect its biodistribution. The mice were anesthetized and imaged by the IVIS Spectrum system (PerkinElmer, USA).

### BLM-induced pulmonary fibrosis and intervention model

For Fer-1 treatment, 6-week-old C57BL/6 male mice were divided into four groups randomly (saline, BLM, BLM + Fer 1, BLM + DMSO), 10 mice in each group. 6 mg/kg of BLM was instilled intratracheally (MKBio Technology Co., Ltd., Shanghai, China). From day 1 to day 21 after BLM treatment, Fer-1 or DMSO (2 mg/kg) was injected into the intervention animals intraperitoneally. Then the mice were euthanized and sacrificed after 22 days.

For liposome treatment, 6-week-old C57BL/6 male mice were divided into four groups randomly (saline, BLM, BLM + UHRF1 liposomal siRNA, BLM + scramble liposomal siRNA), 10 mice in each group. 6 mg/kg of BLM was instilled intratracheally. On day 7 and day 14, UHRF1 or scrambled liposomal siRNAs (1 mg/kg, Ruixi Biological Technology Co., Ltd, Xi An, China) were injected into mice intratracheally. Then the mice were euthanized and sacrificed after 22 days

### Silica-induced pulmonary fibrosis and intervention model

For Fer-1 treatment, 6-week-old C57BL/6 male mice were divided into four groups randomly (saline, SiO_2_, SiO_2_ + Fer 1, SiO_2_ + DMSO), 10 mice in each group. 50 mg/kg of silica particles (Sigma-Aldrich, St. Louis, MO, USA) was instilled intratracheally. From day 1 to day 27 after SiO_2_ treatment, Fer-1 or DMSO (2 mg/kg) was injected into the intervention animals intraperitoneally. Then the mice were euthanized and sacrificed after 28 days.

For liposome treatment, 6-week-old C57BL/6 male mice were divided into four groups randomly (saline, SiO_2_, SiO_2_ + UHRF1 liposomal siRNA, SiO_2_ + scramble liposomal siRNA), 10 mice in each group. 50 mg/kg of silica particles was instilled intratracheally. On day 7, 14, and 21, UHRF1 or scrambled liposomal siRNAs (1 mg/kg, Ruixi Biological Technology Co., Ltd, Xi An, China) were injected into mice intratracheally. Then the mice were euthanized and sacrificed after 28 days.

### Histopathology

The mouse lungs were inflated with a neutral buffered formalin solution overnight and embedded in paraffin. Then, the sections were stained with hematoxylin and eosin (H&E), Masson’s trichrome stain, Sirius red, and immunohistochemistry analysis for UHRF1 expression. Panoramic Scanning Electron Microscope was used to scan the slides. Fibrosis was scored using the Ashcroft scoring method.

### Hydroxyproline content assay

The lung hydroxyproline content was measured by hydroxyproline content assay (A030-2, Jiancheng Bioengineering Institute, Nanjing, China) according to the manufacturer’s protocol. The experiment was replicated three times in the study.

### Serum biochemical determinations

Mice blood was obtained and preserved at 4 °C overnight, then centrifuged at 3500 rpm for 5 min to obtain serum. Alanine aminotransferase/pyruvate transaminase (ALT/GPT) and aspartate aminotransferase (AST), the serum levels of creatinine (Scr), blood urea nitrogen (BUN) and creatine kinase-MB (CK-MB) were measured in serum by using the kits from Jiancheng Bioengineering Institute, Nanjing, China. The experiments were replicated three times in the study.

### Cell culture and treatment

The C57BL/6 mouse primary AEC2 cells and primary murine lung fibroblasts (PLFs) were isolated. The lung epithelial cells A549 and human monocytic cell THP-1 were purchased from American Type Culture Collection (ATCC, Manassas, VA, USA). All cell lines were authenticated using STR profiling within the last 2 years, and mycoplasma-free cells were used in experiments. AEC2 and PLFs cells were maintained in Dulbecco’s modified Eagle’s medium (DMEM, Life Technologies/Gibco, Grand Island, NY, USA); while A549 and THP-1 cells were maintained in RPMI Medium 1640 basic (1640, Life Technologies/Gibco, Grand Island, NY, USA). All of the culture media contained 10% fetal bovine serum (BISH1475, Biological Industries) and antibiotics (penicillin and streptomycin, Life Technologies/Gibco, Gaithersburg, MD). All the cells were cultured at 37 °C with 5% CO_2_.

PMA (Sigma–Aldrich) was used to treat THP-1 cells into macrophages. For all the experiments analysis, AEC2 and THP-1 were treated with 150 μg/ml Silicon dioxide (SiO_2_) (Sigma-Aldrich, St. Louis, MO, USA) together with different intervention factors, according to specific experimental needs. PLFs cells were treated with 5 ng/ml TGF-β1 (Sigma-Aldrich) together with other intervention factors. Erastin (E7781) and Fer-1 (SML0583) were obtained from Sigma-Aldrich. Deferoxamine (DFO, HY-B1625) was obtained from MedChemExpress. GPX4 plasmid from Generay Biotech (Shanghai, China), and FSP1 plasmid from Origene, Inc (Origene, Rockville, MD, USA), UHRF1 inhibitor NSC232003, ferroptosis inhibitors ferrostain-1, a pan-caspase inhibitor ZVAD-FMK, a potent necroptosis inhibitor necrostatin-1, and the DNA methyltransferase inhibitor 5-AZA-dC were phased from MedChemExpress.

UHRF1 siRNA and control siRNA were synthesized by GenePharm (Shanghai, China). Cells were transfected using riboFECTCP Reagent (Ribobio, Guangzhou, China) according to the manufacturer’s protocol. The sequences of UHRF1 siRNA were shown as follows: UHRF1, sense: AGACGGAAUUGGGGCUGUATT; antisense: UACAGCCCCAAUUCCGUCUTT;

### Immunostaining assay

Adherent cells were fixed with 4% paraformaldehyde for 30 min, washed with PBS and blocked with 10% BSA for 1 h at room temperature. Then stained with a primary antibody against α-SMA (Abcam, ab32575, 1:200), an antibody against GPX4 (Abcam, ab125066, 1:200), and an antibody against FSP1 (ABclonal, A12128, 1:200) overnight at 4°C, and incubated with Cy3-conjugated or FITC-conjugated goat or mouse anti-rabbit antibody (1:200, Beyotime Institute of Biotechnology, Shanghai, China) for 1 h. The nuclear was then stained with 6-diamidino-2-phenylindole (DAPI). Fluorescent images were acquired with the fluorescence microscope (Olympus, Tokyo, Japan). The experiment was replicated three times in the study.

### Western blot and antibodies

The western blot was performed as described previously [32]. The experiment was replicated three times in the study. Original bands were presented in Supplemental Material.

Primary antibodies: antibody against UHRF1(Santa Cruz, sc-373750); antibody against GPX4 (Abcam, ab125066); antibody against FSP1(ABclonal, A12128); antibody against Fibronectin (Abcam, ab45688,); antibody against Collagen I (ABclonal, A1352); antibody against α-SMA (Abcam, ab32575); antibody against DNMT1 (Santa Cruz, sc-271729); antibody against UHRF1 (Santa Cruz, H-8, sc-373750); antibody against GAPDH (ABclonal, AC002).

### Quantitative RT‑PCR (qRT‑PCR)

QRT‑PCR was performed as described [32]. The experiment was replicated three times in the study. The primers used in our study were shown in Supplementary Table 1.

### ChIP-qPCR

ChIP assay was performed by using SimpleChIP® Enzymatic Chromatin IP Kit (CST, MA, #9003, USA). Cells were harvested and cross-linked with 1% formaldehyde, followed by the addition of 10×glycine for 5 min. Centrifuge tubes were used to wash, and collect cells. Then, cells were sonicated and immunoprecipitated with IgG, anti-UHRF1, anti-Dnmt1, and anti-Histones antibodies at 4 °C overnight. Then the immunoprecipitates were eluted and reverse cross-linked, followed by DNA fragments purified for PCR amplification. The experiment was replicated three times in the study. Primer sequences are listed in Supplementary Table 2.

### Methylation-specific PCR

The methylation level of the GPX4 and FSP1 promoter regions were detected by the EpiArt DNA Methylation Bisulfite Kit (EM101-01, Vazyme Biotech Co., Ltd., Nanjing, China). The PCR primers for methylation and unmethylation of the GPX4 and FSP1 genes are listed in Supplementary Table 3. The PCR reactions were performed by agarose gel electrophoresis, and the gel was observed with Doc XR + gel imaging system (Bio-Rad).

### Detection of lipid ROS

For fluorescence detection of lipid ROS, cells were stained for 30 min with 10 μM C11-BODIPY (RM02821, ABclonal), washed three times with PBS, and the red and green fluorescence signals in the cells were obtained under a fluorescence microscope. Qualification data was displayed as the ratio of green/red fluorescence intensity in cells. The experiment was replicated three times in the study.

### Lipid peroxidation assay (MDA)

MDA concentration in cell lysates was measured using a Lipid Peroxidation MDA Assay Kit (Beyotime Institute of Biotechnology, Shanghai, China) according to the manufacturer’s instructions. The experiment was replicated three times in the study.

### Glutathione/oxidized glutathione ratio (GSH/GSSG Ratio) measurement

According to the manufacturer’s instructions, the cellular and tissue sample GSH/GSSG ratio was determined using the Total GSH/GSSG Colorimetric Assay Kit (E-BC-K097-M, Elabscience). Both lung tissue and cell GSH/GSSG levels were detected. The experiment was replicated three times in the study.

### Tissue iron content assay

The relative iron concentration in lung tissues was assessed using an Iron Colorimetric Assay Kit (Elabscience, E-BC-K139-M) according to the manufacturer’s instructions. The absorbance was determined at 520 nm using a microplate reader.

### Measurement of Intracellular Fe^2+^

A FerroOrange fluorescent probe(MX4559-48UG, Serve Life Science) was used for the detection of intracellular Fe^2+^. The cells were rinsed and incubated with 1 µM FerroOrange for 1 h. The cells were observed under a fluorescence microscope after washing. Then, Fe^2+^ fluorescence intensity was quantified with ImageJ software. The experiment was replicated three times in the study.

### Flow cytometry

Dead cells were detected using Annexin V-FITC Apoptosis Detection Kit (Dojindo, Shanghai, China). Briefly, cells were digested and washed twice with PBS. 100 μl prepared 1 × Annexin V Binding Solution was used to prepare cell suspension. Then the cells were stained with 5 μl PI working solution for 15 min in the dark. Finally, the stained cells were analyzed with the FACS Caliber Flow Cytometer (Becton Dickinson, Franklin Lakes, NJ, USA). The experiment was replicated three times in the study.

### RNA-seq

The integrity and purity of RNA were checked prior to library preparations. A NEBNext® UltraTM RNA Library Prep Kit for Illumina was used according to the manufacturer’s recommendations to create the sequencing libraries, which were then sequenced in 150-bp paired-end reads using an Illumina Hiseq platform (Shanghai Biotree Biotech Co., LTD, China).

### Murine AEC2 cells 3D culture

Primary cells were mixed with 1:1 growth factor–reduced Matrigel (BD Biosciences); 90 μl matrigel was placed in a 24-well 0.4-μm Transwell insert (Falcon). Then 5 × 10^3^ AEC2s and 1 × 10^5^ primary fibroblast cells were seeded on the matrigel. 500 μl MTEC/Plus was placed in the lower chamber, and the medium was changed every other day. Also, ROCK inhibitor (Y0503; Sigma-Aldrich) was added to the medium for the first 2 days. For paraffin embedding, the Matrigel disc was removed and then dehydrated by using ethanol, and transitioned into paraffin as former instructed. And for passaging experiments, the cell spheres were dissociated by 60 μl Dispase (catalog #354235, BD Biosciences) and incubation for 30 min. Cells were washed and then incubated in 0.05% Trypsin-EDTA for 30 min at 37 °C.

### Statistical analysis

Comparisons between groups were performed using Graph Pad Prism (version 6.01) software (GraphPad Software Inc., San Diego, CA, USA). Student’s *t*-test was used for statistical analysis between two groups, while one-way analyses of variance followed by Tukey’s multiple comparisons test were used for more than two groups. The data were presented as mean ± SD. Moreover, the *P* value <0.05 was considered with statistical significance.

## Supplementary information

Reproducibility checklist

Supplementary Figures

Supplementary Tables

Full and uncropped western blots

## Data Availability

Source data are provided with supplementary files and in www.figshare.com, 10.6084/m9.figshare.21314022. The data that support the findings of this work are available from the corresponding author on request. There are no restrictions on data availability in the current work.
