# Supplementary material for: UHRF1-mediated ferroptosis promotes pulmonary fibrosis via epigenetic repression of GPX4 and FSP1 genes
Source: Cell Death Dis. 2022 Dec 24;13(12):1070. doi: 10.1038/s41419-022-05515-z (PMC9789966; doi:10.1038/s41419-022-05515-z)
Supplement: Supplementary file 2 — Supplementary Figures [file 41419_2022_5515_MOESM2_ESM.docx]

**
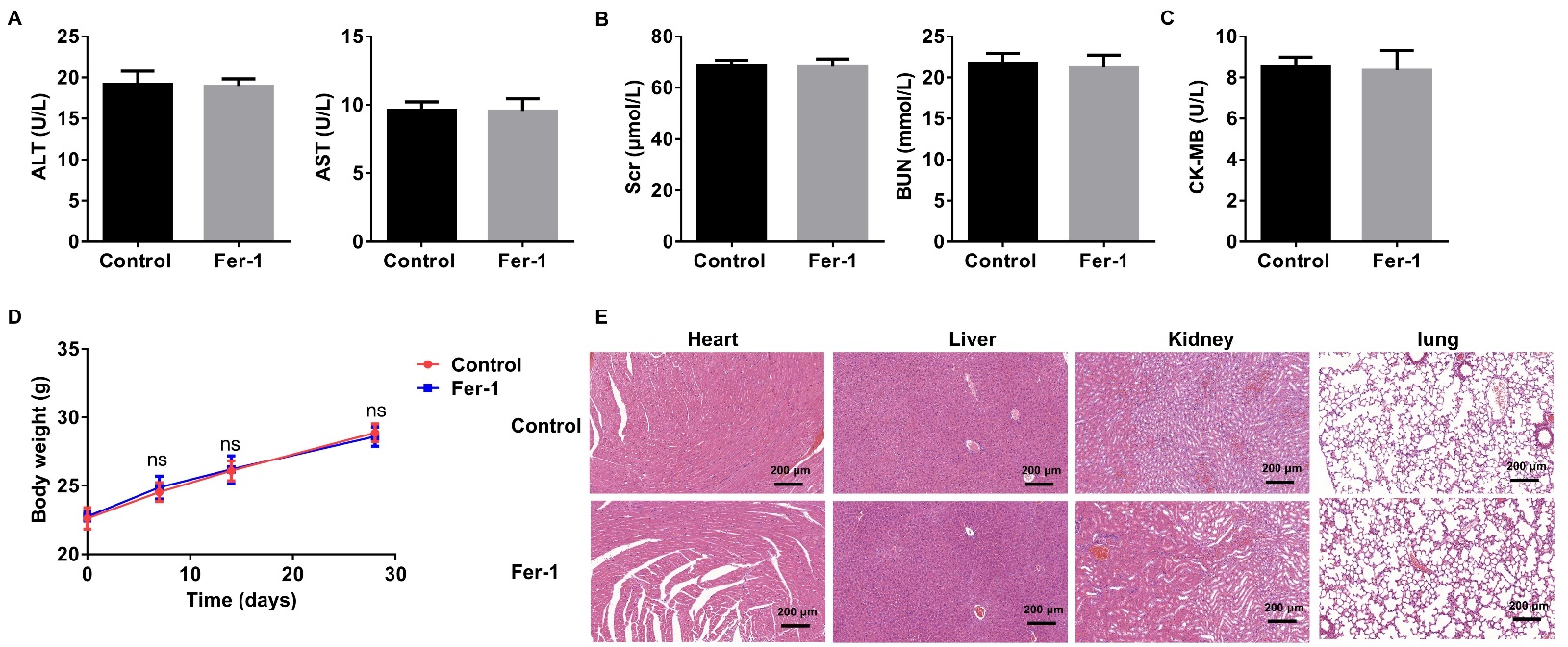
**

**Supplementary Figure 1** The toxicity effect of Fer-1 in vivo. The 6-week C57BL/6 mice were treated with or without Fer-1 for 28 days. **A~C** The levels of alanine aminotransferase/pyruvate transaminase (ALT/GPT) and aspartate aminotransferase (AST), the serum levels of creatinine (Scr), blood urea nitrogen (BUN) and creatine kinase-MB (CK-MB) in the mice serum. **D** The body weight of the mice in the control and the Fer-1 group. **E** H&E staining of the heart, liver, kidney, and lung in the control and the Fer-1 treatment groups.


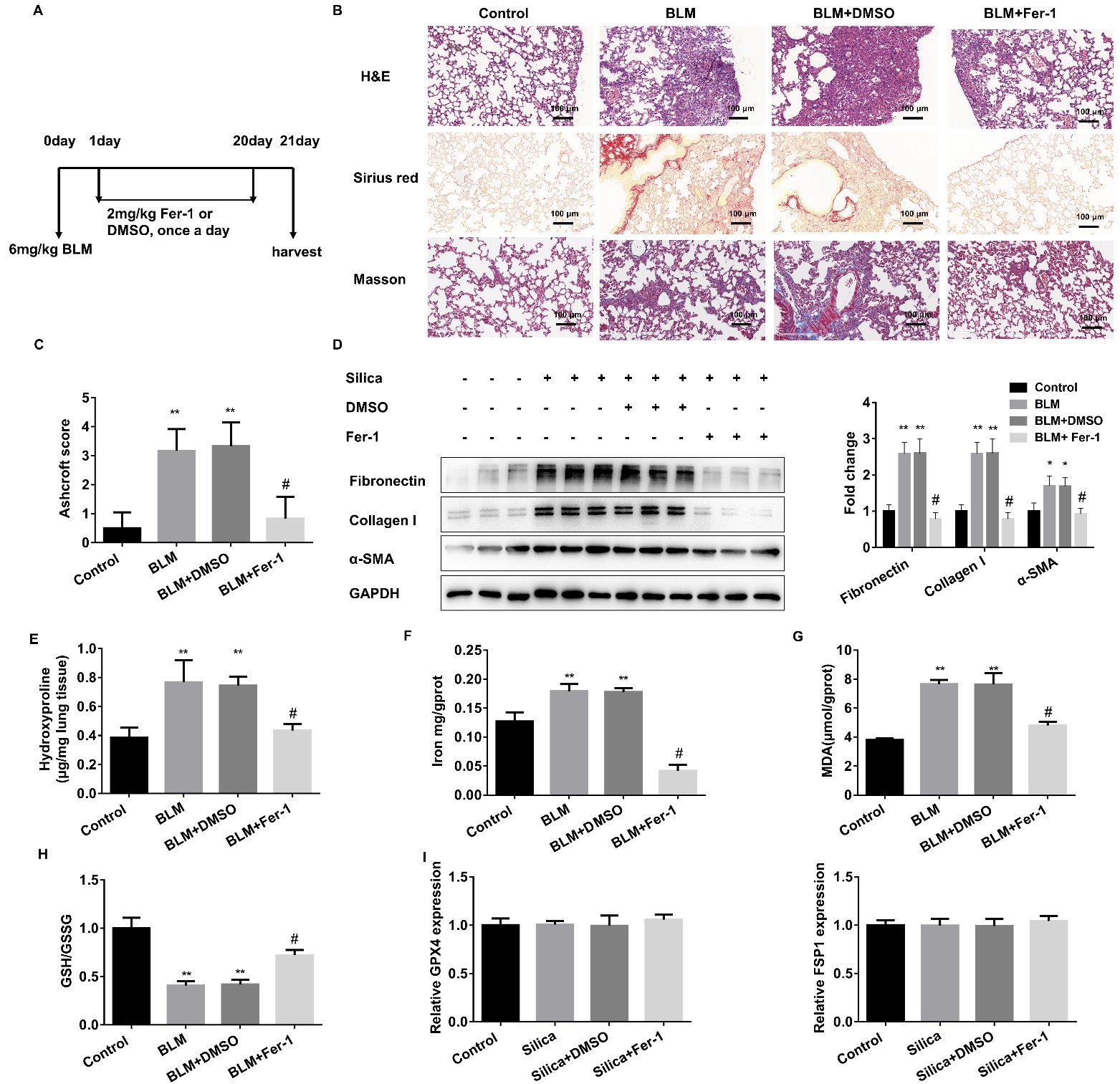


**Supplementary Figure 2** Ferroptosis inhibitor alleviates BLM-induced mouse PF. **A** Schematic diagram of Fer-1 or DMSO-treated mouse BLM-induced PF model. **B** Representative hematoxylin/eosin (HE), Sirius red, and Masson staining of lung tissues from each group of C57BL/6 mice sacrificed on day 21 (scale bars, 100 µm). **C** Ashcroft score of mice from each group. **D** The protein levels of Fibronectin, Collagen I, and α-SMA in lung tissues were detected by western blot and qualified (means ± SD, n = 3). **E** The levels of hydroxyproline content were determined at 550 nm and expressed as micrograms per mg of lung tissues, determined by the hydroxyproline content assay kit. **F** The labile iron concentration of lung tissues was assessed using an Iron Colorimetric Assay Kit. **G** MDA concentration lung tissues was measured using a Lipid Peroxidation MDA Assay Kit. **H** GSH/GSSG ratio of lung tissues. **I** qRT-PCR of mouse lung GPX4 and FSP1 mRNA levels. ^*^*P* < 0.05, ^**^*P* < 0.01 versus control group, ^#^*P* < 0.01 versus BLM+DMSO treated group


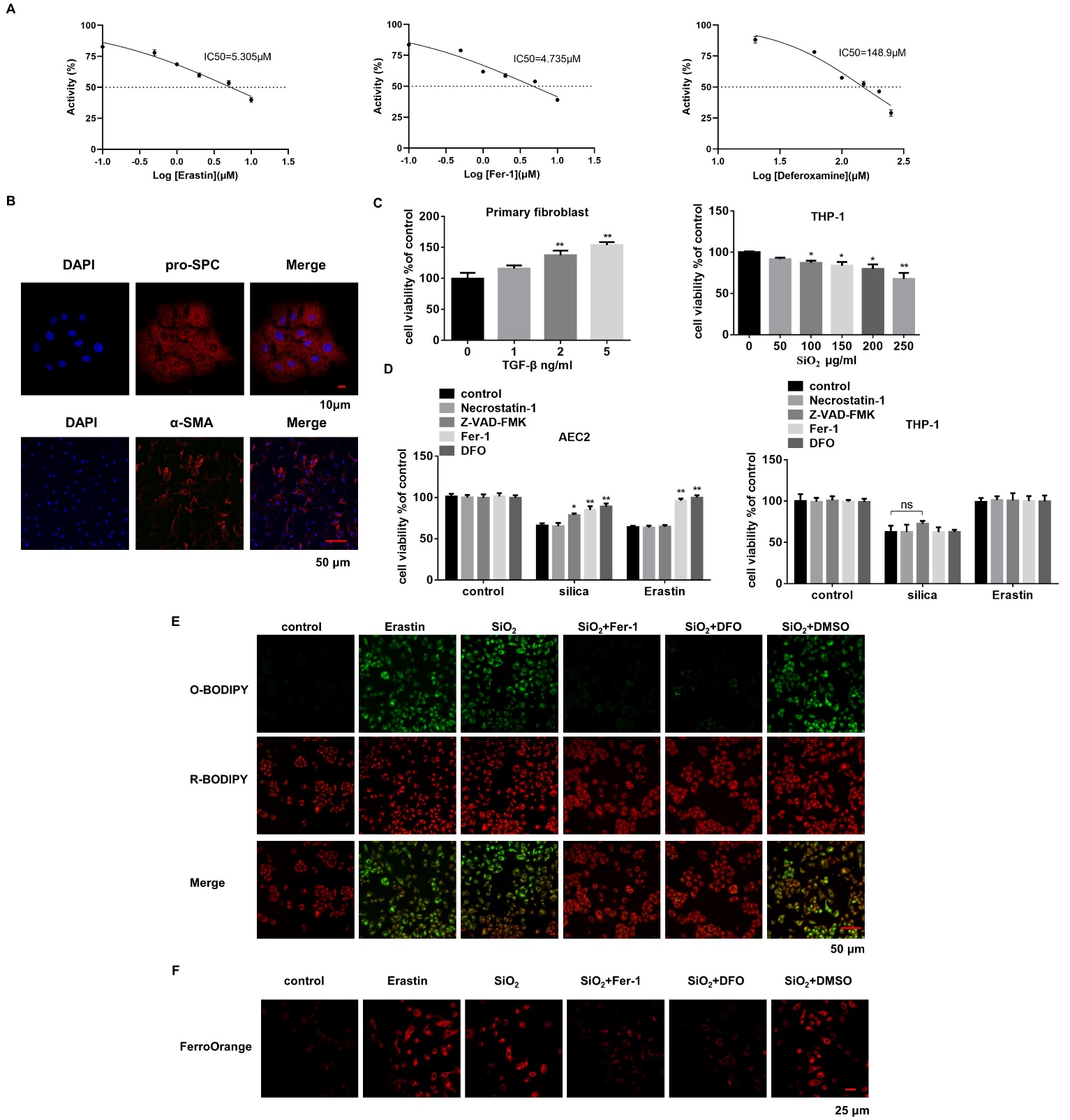


**Supplementary Figure 3** Ferroptosis mainly occurs in AEC2 cells otherwise macrophages and fibroblasts during PF. **A** IC-50 value of eratsin, Fer-1, and DFO in primary AEC2 cells. **B** Representative immunofluorescence staining of pro-SPC in primary murine AECs (scale bars, 10 µm) and α-SMA in primary murine fibroblasts (scale bars, 50 µm). **C** CCK8 detected mouse primary fibroblast and THP-1-induced macrophage cell viability after treated with different doses of TGF-β1 and SiO_2_ (^*^*P* < 0.05, ^**^*P* < 0.01). **D** CCK8 detected control, silica-treated, and ferroptosis inducer erastin-treated mouse primary AEC2 and THP-1-induced macrophage cell viability, after co-treated with necroptosis inhibitor necrostatin-1, apoptosis inhibitor Z-VAD-FMK, and ferroptosis inhibitor Fer-1. **E** Representative images of C11-BODIPY in A549 cells (*red*: reduced C11-BODIPY, *green*: oxidized C11-BODIPY; scale bars, 50 µm). **F** Intracellular Fe^2+^ was detected with the FerroOrange probe, and representative images of A549 cells were shown (*red*: FerroOrange-stained Fe^2+^; scale bars, 25 µm; left panel).


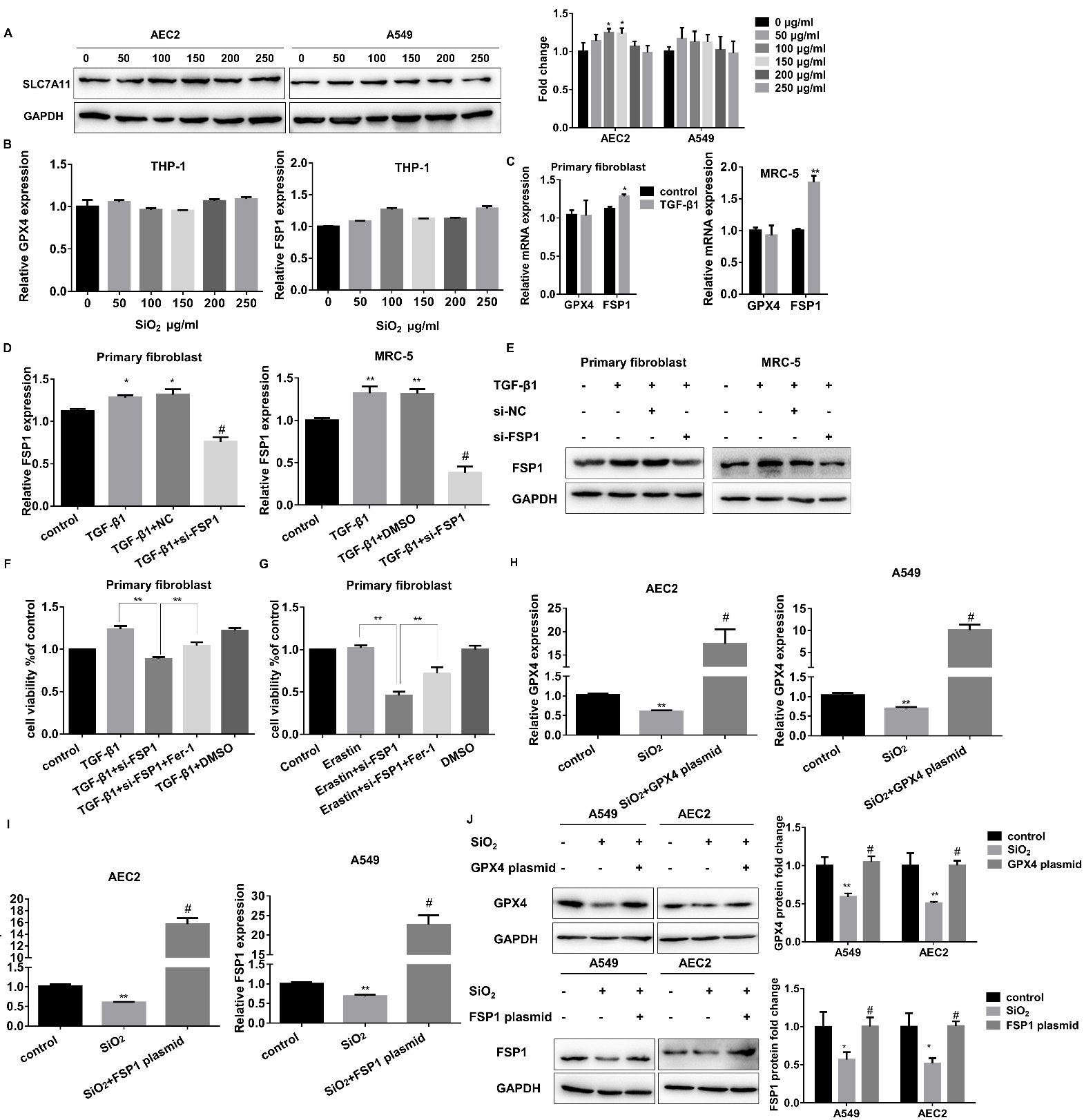


**Supplementary Figure 4** FSP1 blockage decreased cell viability in TGF-β1 activated lung fibroblasts via ferroptosis. **A** Representative immunoblotting of SCL7A11 in 0, 50, 100, 150, 200, and 250 µg/mL SiO_2_ treated primary AEC2 and A549 cells (left panel), means ± SEM of three independent experiments (right) are shown. qRT-PCR of GPX4/FSP1 mRNA levels in SiO_2_ treated THP-1 macrophages (**B**) and TGF-β1-treated lung fibroblasts (**C**) (^*^*P* < 0.05, ^**^*P* < 0.01). (**D**) RNA and (**E**) protein levels of FSP1 in TGF-β1 together with FSP1 siRNA -treated lung fibroblasts. CCK8 cell viability of control and TGF-β1-treated (**F**) or erastin-treated (**G**) lung fibroblasts together with FSP1 siRNA and Fer-1 or DMSO. qRT-PCR of GPX4 (**H**) and FSP1 (**I**) mRNA levels in SiO_2_ with or without plasmid treated primary murine AEC2 and A549 cells. **J** Immunoblotting of GPX4 and FSP1 protein levels in SiO_2_ with or without plasmid treated AEC2s, means ± SEM of three independent experiments (right) are shown.


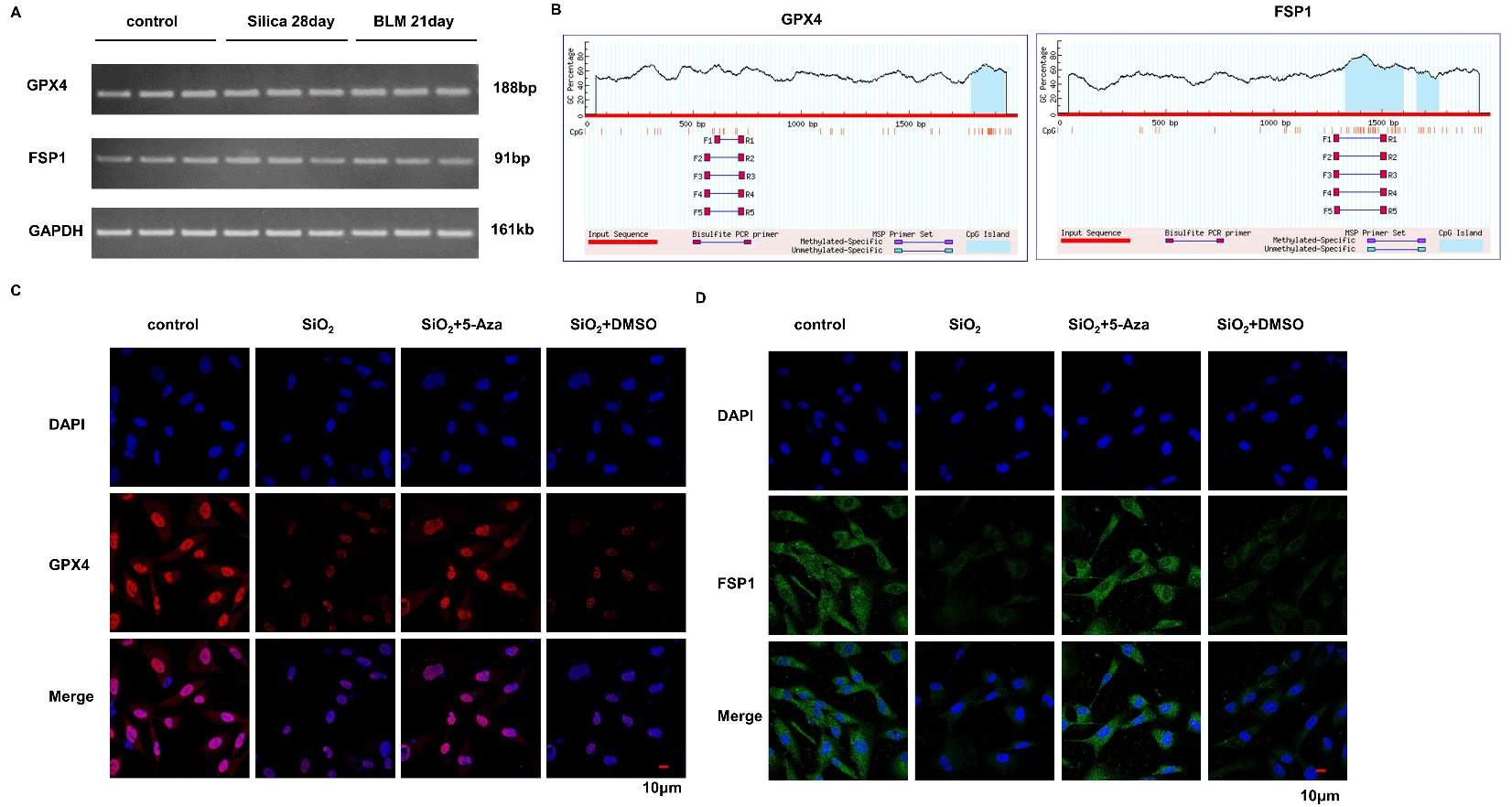


**Supplementary Figure 5** DNA methylation regulates GPX4 and FSP1 expression in AEC2s. **A** PCR of GPX4 and FSP1 genomic DNA levels in control, silica-treated 28-day, and BLM-treated 21-day mouse lung primary AEC2 cells. **B** DNA methylation sites prediction of GPX4 and FSP1 gene promoters. Control, SiO_2_-treated, SiO_2_ together with 5-Aza or DMSO-treated A549 cells. Representative immunofluorescence staining of GPX4 (**C**) and FSP1 (**D**) in A549 cells.


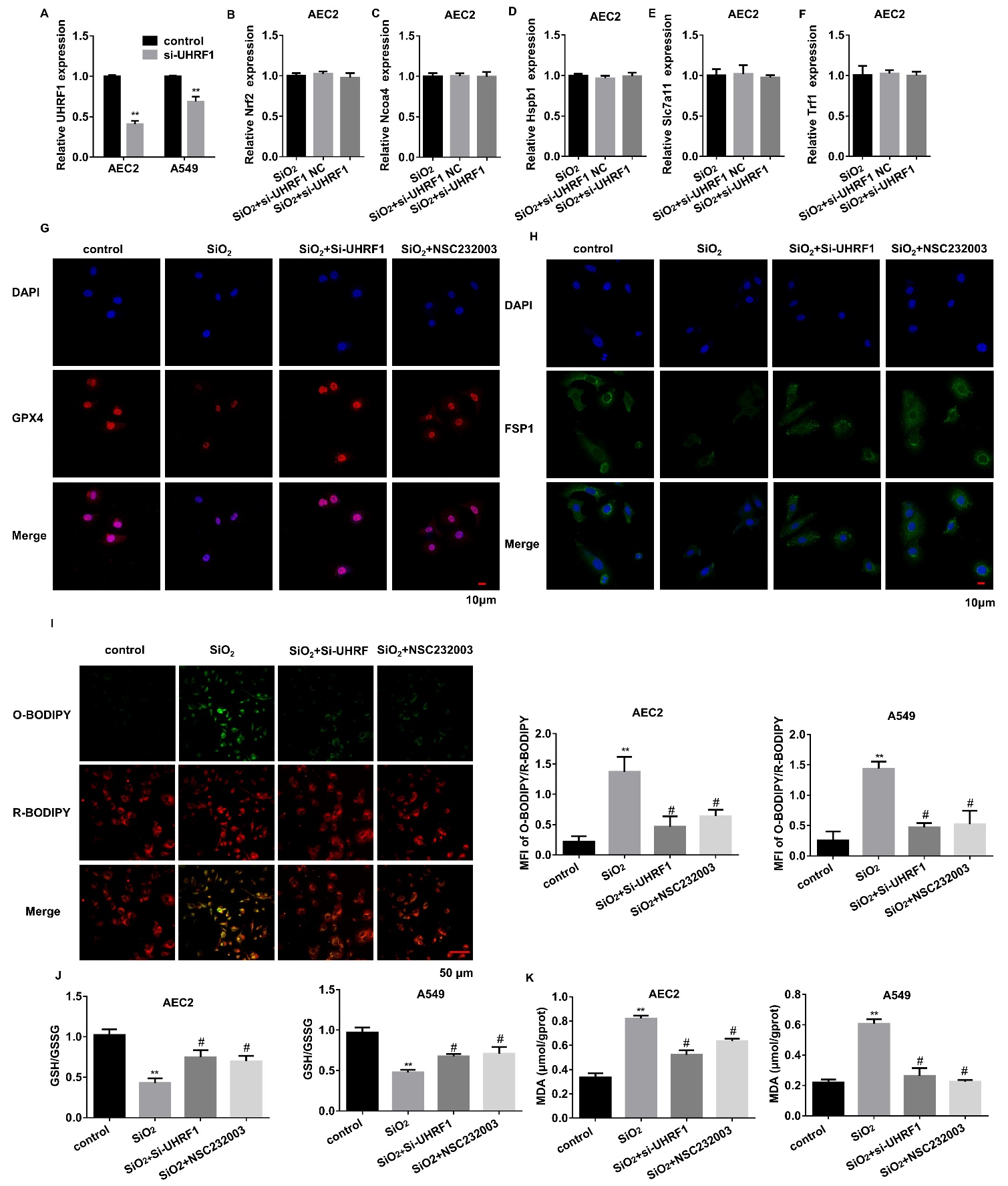


**Supplementary Figure 6** UHRF1 regulates GPX4/FSP1 via DNA methylation. **A** qRT-PCR of UHRF1 siRNA knockdown efficiency. **B~F** mRNA levels of Nrf2, Ncoa4, Hspb1, Slc7a11, and Trf1 levels were measured individually by qRT-PCR in primary AEC2 cells. Then, control, SiO_2_-treated, SiO_2_ together with UHRF1 siRNA or UHRF1 inhibitor NSC232003 treated A549 cells. Representative immunofluorescence staining of GPX4 (**G**) and FSP1 (**H**) in A549 cells. **I** Representative images of C11-BODIPY in treated-primary AEC cells (*red*: reduced C11-BODIPY, *green*: oxidized C11-BODIPY; scale bars, 50 µm; left panel), and quantification of C11-BODIPY fluorescence in primary AEC2 and A549 cells (right panel). **K** GSH/GSSG ratio of primary AEC2 and A549 cells. **J** MDA concentration in cell lysates from primary AEC2 and A549 cells was measured using a Lipid Peroxidation MDA Assay Kit. ^**^*P* < 0.01 versus control group, ^#^*P* < 0.01 versus SiO_2_-treated group.


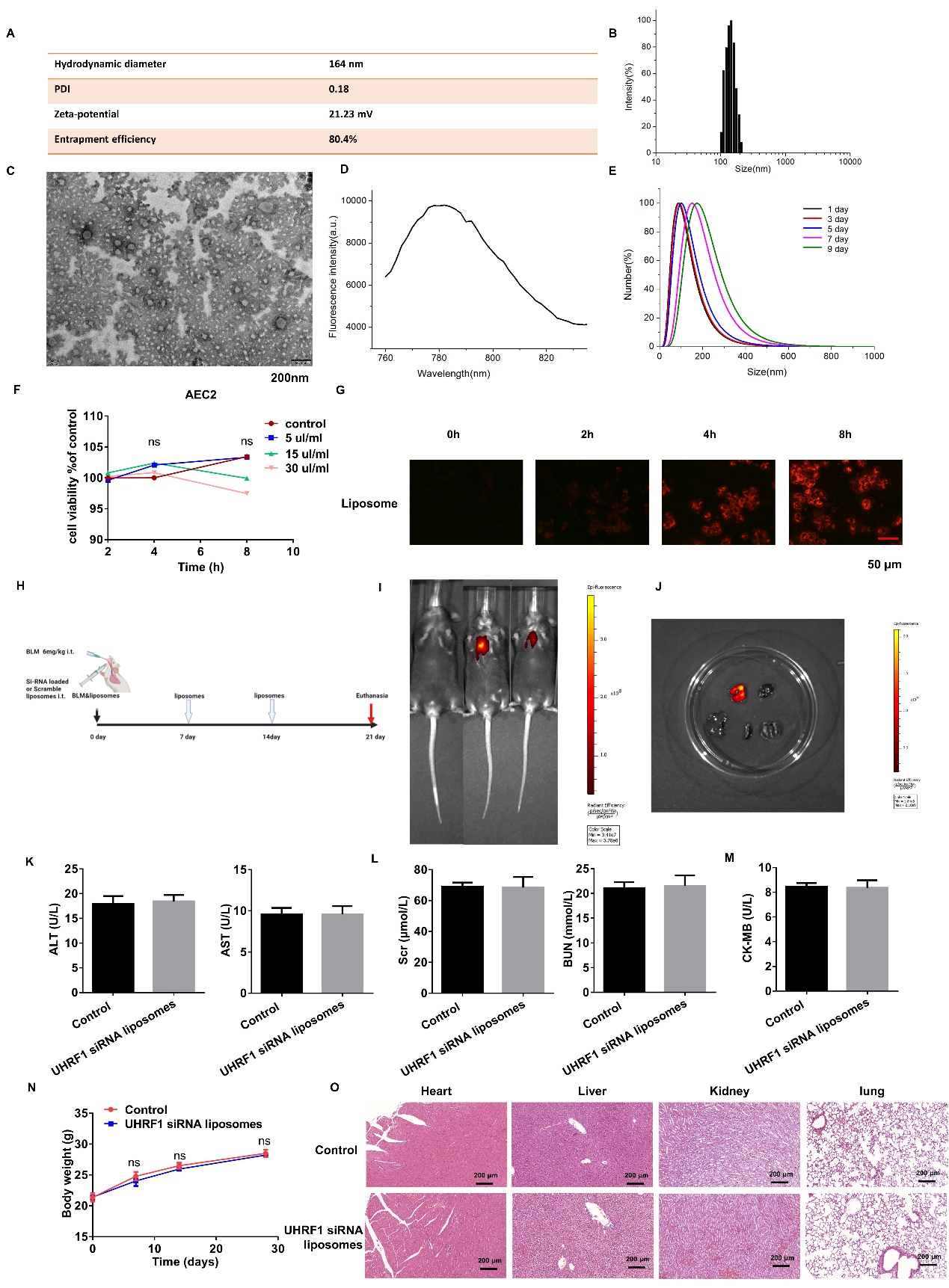


**Supplementary Figure 7** UHRF1 siRNA liposome character and biodistribution. **A** The hydrodynamic diameter, PDI and zeta potential of the liposomes (blank or siRNA-loaded) were measured by DLS. **B** Hydrodynamic diameter distribution of siRNA-loaded liposomes. **C** Representative TEM image of siRNA-loaded liposomes. **D** Wavelength of DiR fluorescence. **E** Stability of siRNA-loaded liposomes after 1~9 days. **F** CCK8 detected cell toxicity of liposome in ACE2 cells. **G** Representative fluorescence of UHRF1 liposome-transfected ACE2 cells in 8 h. **H** Schematic diagram of UHRF1 siRNA liposome or scramble liposome-treated together with BLM mouse experiment. **I** Representative IVIS images of 28 day mouse after the administration of DiR-labeled UHRF1 siRNA liposomes and scramble liposomes. **J** Representative IVIS images of lung, heart, liver, spleen, and kidney in UHRF1 siRNA liposomes-treated 28 day mouse. IVIS: in vivo imaging system. The 6-week C57BL/6 mice were treated with or without UHRF1 siRNA liposomes for 28 days. **K~M** The levels of ALT/GPT and AST, Scr, BUN and CK-MB levels in the mice serum. **N** The body weight of the mice in the control and the UHRF1 siRNA-loaded liposomes group. **E** H&E staining of the heart, liver, kidney, and lung in the control and the UHRF1 siRNA liposomes treatment groups.


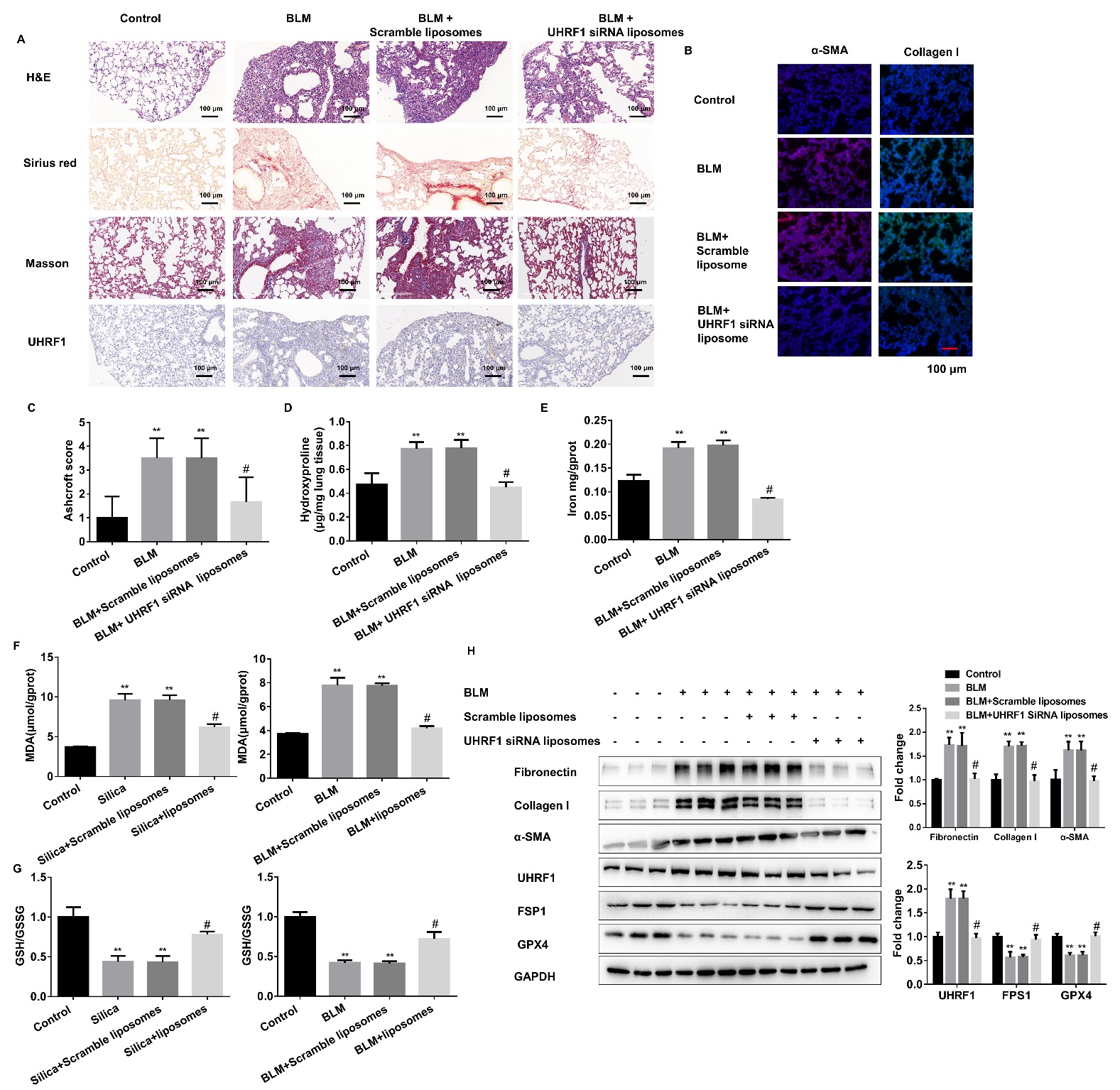


**Supplementary Figure 8** UHRF1 liposomal siRNA reverse BLM-induced PF progression in vivo. BLM were injected intra-tracheally on day 0. UHRF1 siRNA liposomes or scramble liposomes were injected intra-tracheally on day 0, 7, 14, then mice were sacrificed in day. 21. **A** Representative HE, Sirius red, UHRF1 IHC and Masson staining of lung tissues from each group of C57BL/6 mice sacrificed on day 21 (scale bars, 100 µm). **B** Representative immunofluorescence staining of α-SMA and Collogen I from mouse lung tissues (scale bars, 100 µm). **C** Ashcroft score of mice from each group. **D** The levels of hydroxyproline content were determined at 550 nm and expressed as micrograms per mg of lung tissues, determined by the hydroxyproline content assay kit. **E** The labile iron concentration of lung tissues was assessed using an Iron Colorimetric Assay Kit. In both sililica or BLM+UHRF1 liposomes-treated mice, (**F**) MDA concentration in lung tissues and **(G)** GSH/GSSG ratio of lung tissues were measured. **H** The protein levels of Fibronectin, Collagen I, α-SMA in lung tissues, and UHRF1, FSP1, GPX4 in primary AEC2 cells from each group were detected by western blot and qualified. ^**^*P* < 0.01 versus control group, ^#^*P* < 0.01 versus BLM+scramble liposome group
