# Supplementary material for: UHRF1-mediated ferroptosis promotes pulmonary fibrosis via epigenetic repression of GPX4 and FSP1 genes
Source: Cell Death Dis. 2022 Dec 24;13(12):1070. doi: 10.1038/s41419-022-05515-z (PMC9789966; doi:10.1038/s41419-022-05515-z)
Supplement: Supplementary file 3 — Supplementary Tables [file 41419_2022_5515_MOESM3_ESM.docx]

| Genn Name | Forward primer (5'-3') | Reverse primer (3'-5') |
| --- | --- | --- |
| UHRF1 (Homo) | AACTGCTTTGCTCCCATCA | TCTTGCCACCCTTGACATT |
| GPX4 (Homo) | GAGGCAAGACCGAAGTAAACTAC | CCGAACTGGTTACACGGGAA |
| FSP1 (Homo) | GGCCAACATCGTCAACTCT | GCCCACACCGTCATTTCT |
| GAPDH (Homo) | CCTTCCGTGTCCCCACT | GCCTGCTTCACCACCTTC |
| Uhrf1 (Mus) | CCAAGAATGTCCGTGCTC | GGTTTGGCGCTTCCTAC |
| Gpx4 (Mus) | TGTGCATCCCGCGATGATT | CCCTGTACTTATCCAGGCAGA |
| Fsp1 (Mus) | AGAACCGGATGGTGTTGCTAC | CACCTCGTTAAACTTGCCAGG |
| GAPDH (Mus) | TGTTTCCTCGTCCCGTAGA | ATCTCCACTTTGCCACTGC |

Supplementary Table 1

Primer Sequence for RT-qPCR

Supplementary Table 2

| Primers | Sequence (5'-3') |
| --- | --- |
| UHRF1 -ChIP-F | CGCGGAACAGTCTTGTGA |
| UHRF1 -ChIP-R | GGCCCCTTCTTGGTCATT |
| GPX4 -ChIP-F | TGAGTGTGGTTTGCGGAT |
| GPX4 -ChIP-R | CTCCCTGGCTCCTGCTT |
| FSP1-ChIP-F | TGCTGTGGTACTGGGTGAG |
| FSP1-ChIP-F | GAGGAAGGGCTGGCTGT |

Primer Sequence for ChIP-qPCR

Supplementary Table 3

| Primers | Sequence (5'-3') |
| --- | --- |
| GPX4(M) | F: GGGTGGTTTTATAGTTTGGATATC |
|  | R: ACAAATCCTTCTCTATCACCTATCG |
| GPX4(U) | F: GGGGTGGTTTTATAGTTTGGATATT |
|  | R: AACAAATCCTTCTCTATCACCTATCAA |
| FSP1(M) | F: GTTTTTGAGATGTGGGTTTGTATAC |
|  | R: TCCTTTACTTCCTACTCCCGAATA |
| FSP1(U) | F: TTTGAGATGTGGGTTTGTATATGTT |
|  | R: TTTCCTTTACTTCCTACTCCCAAATA |

Primer Sequence for MSP

M: methylation; U: unmethylation
