# Supplementary material for: UHRF1-mediated ferroptosis promotes pulmonary fibrosis via epigenetic repression of GPX4 and FSP1 genes
Source: Cell Death Dis. 2022 Dec 24;13(12):1070. doi: 10.1038/s41419-022-05515-z (PMC9789966; doi:10.1038/s41419-022-05515-z)
Supplement: Supplementary file 4 — Full and uncropped western blots [file 41419_2022_5515_MOESM4_ESM.pptx]

## Slide 1
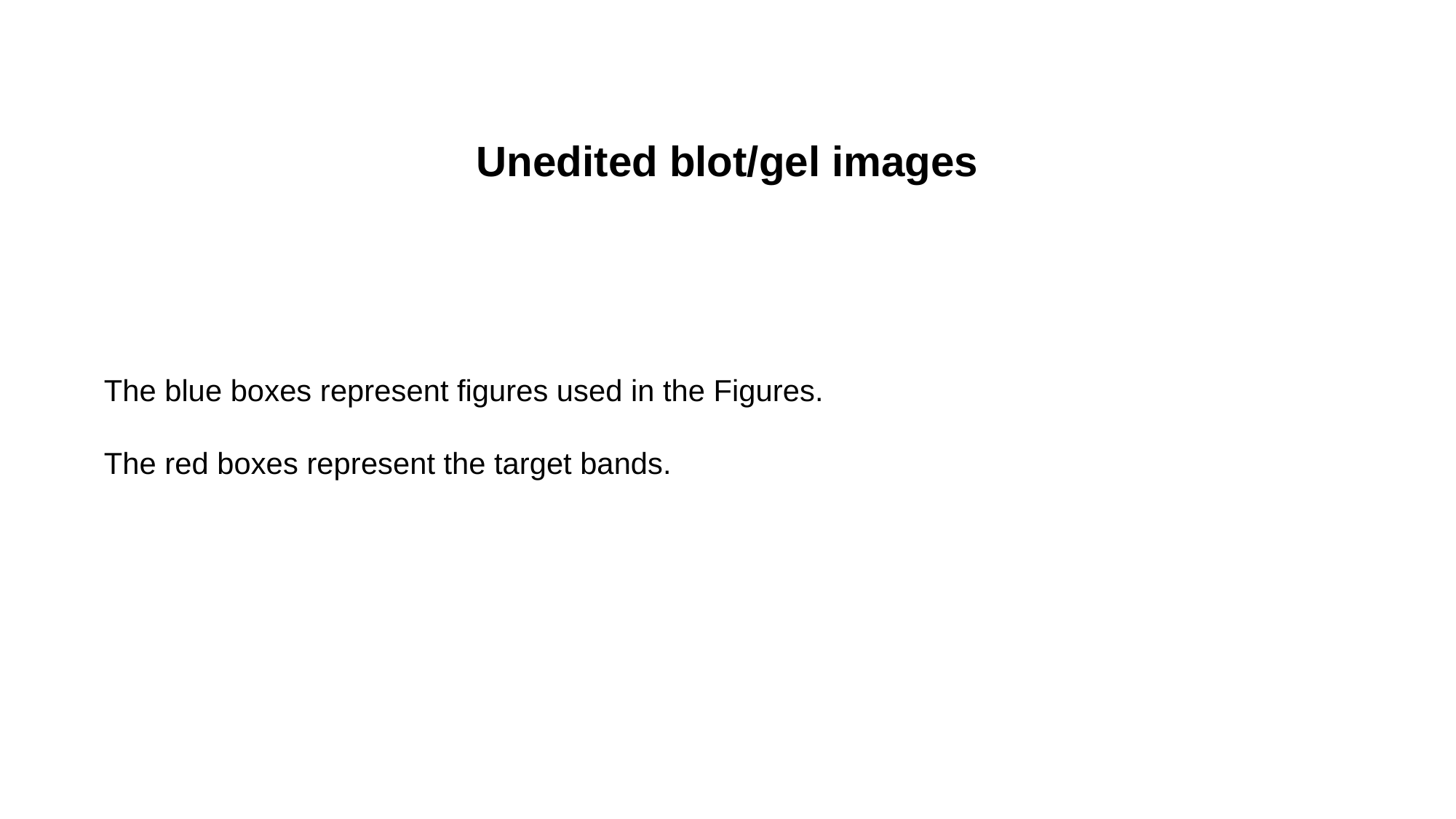

Unedited blot/gel images
The blue boxes represent figures used in the Figures.
The red boxes represent the target bands.

## Slide 2
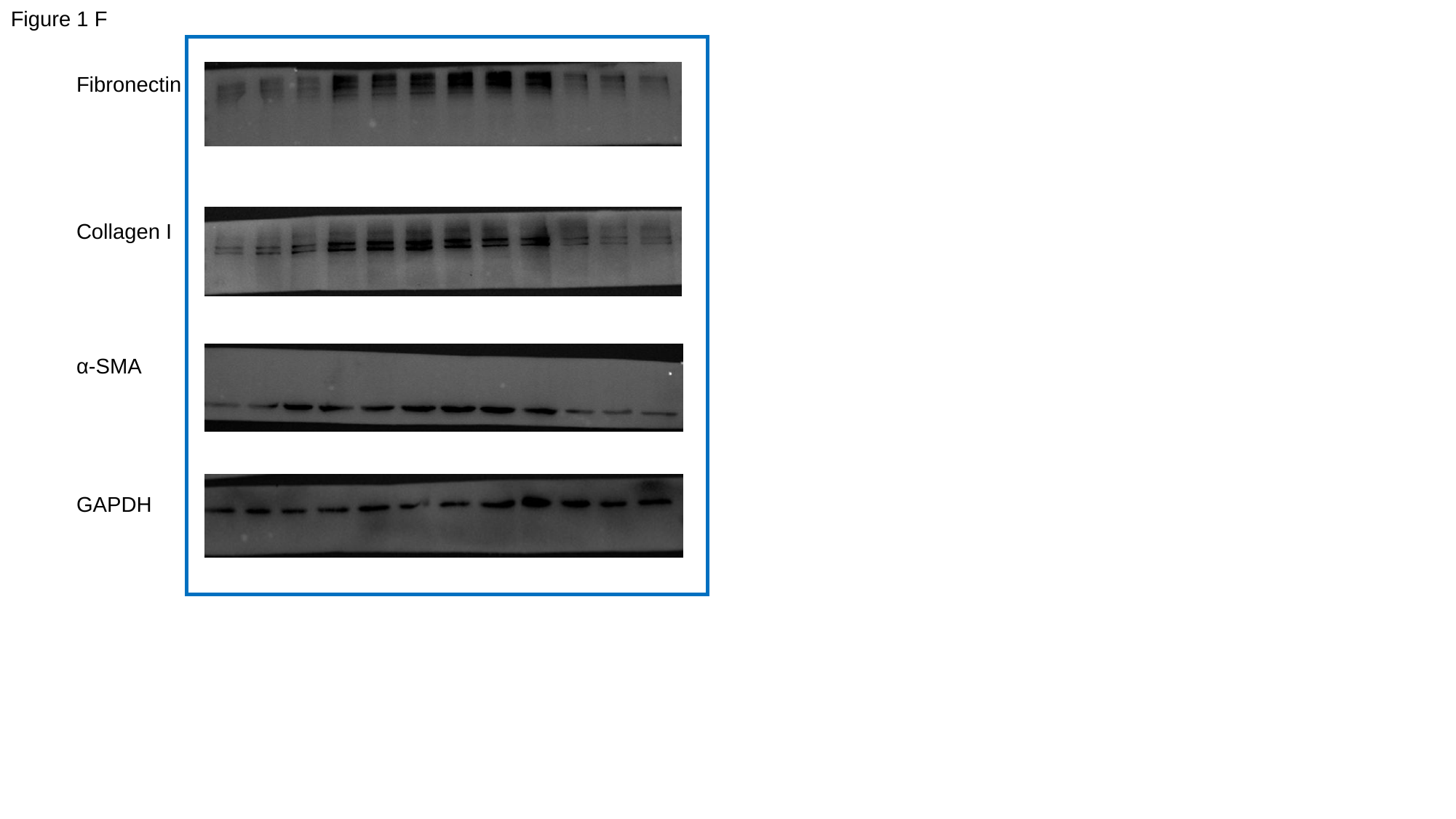

Figure 1 F
Fibronectin
Collagen I
α-SMA
GAPDH

## Slide 3
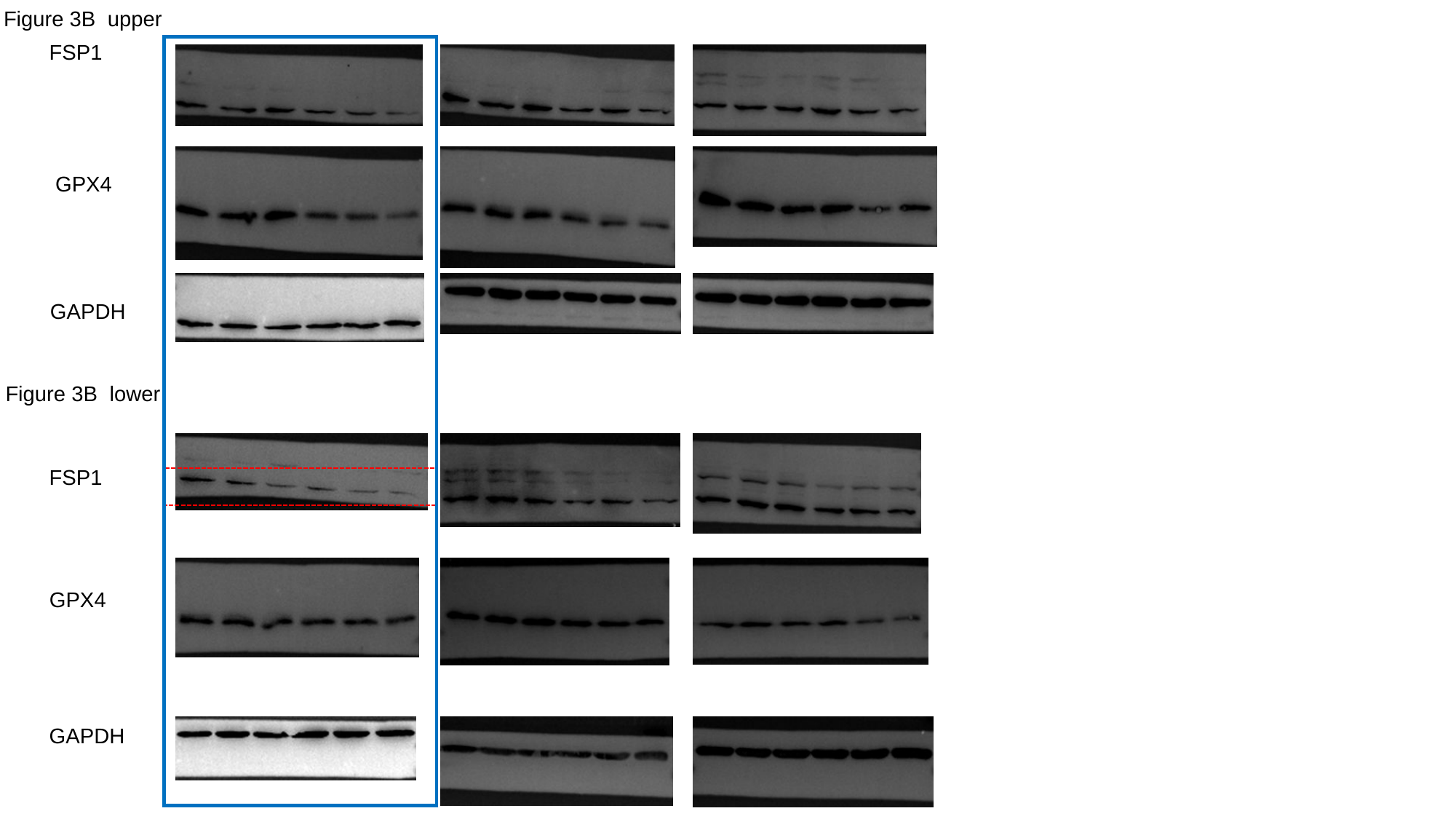

Figure 3B upper
FSP1
 GPX4
GAPDH
Figure 3B lower
FSP1
GPX4
GAPDH

## Slide 4
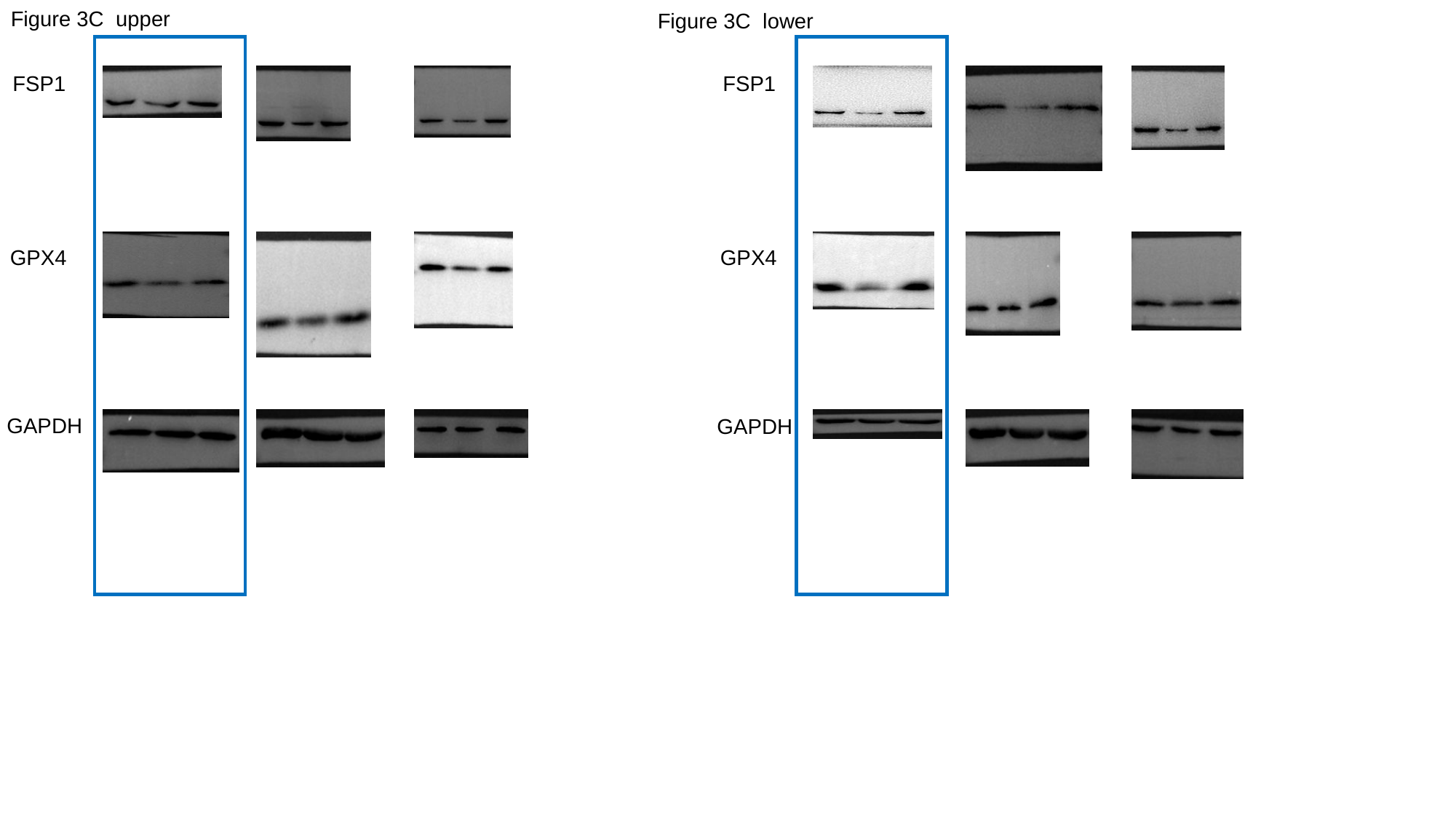

Figure 3C upper
Figure 3C lower
FSP1
FSP1
 GPX4
 GPX4
GAPDH
GAPDH

## Slide 5
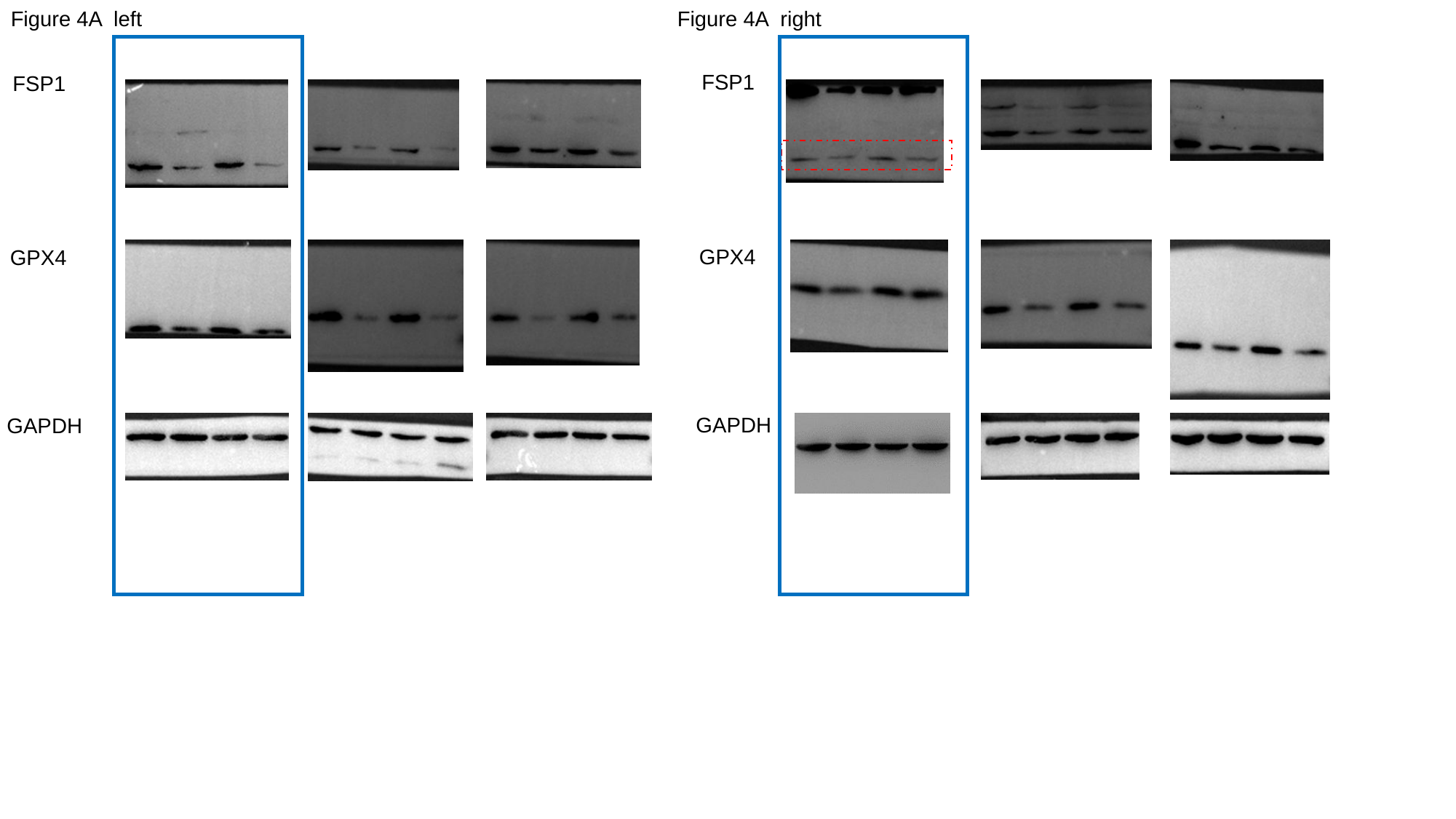

Figure 4A left
Figure 4A right
FSP1
FSP1
 GPX4
 GPX4
GAPDH
GAPDH

## Slide 6
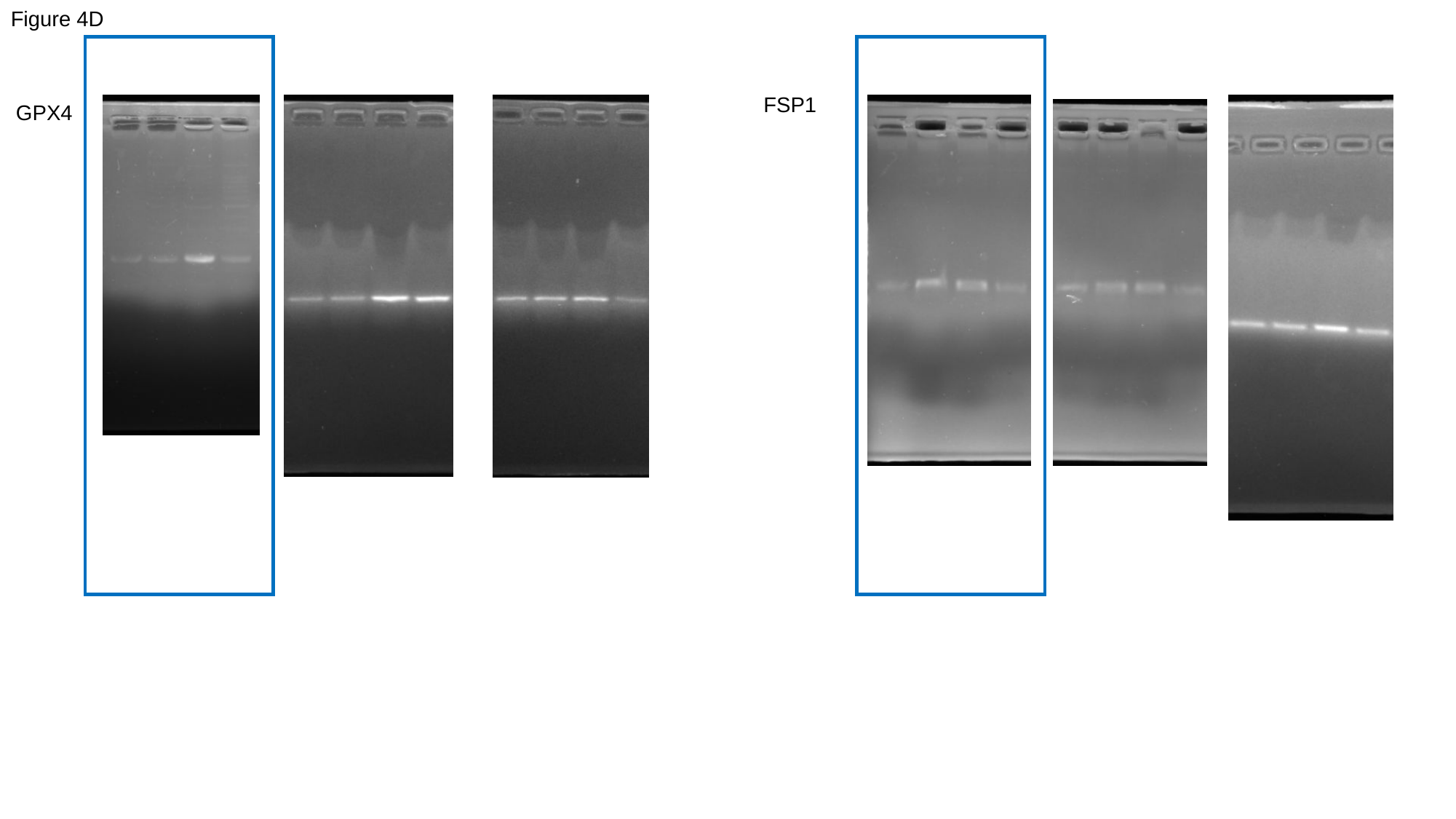

Figure 4D
FSP1
GPX4

## Slide 7
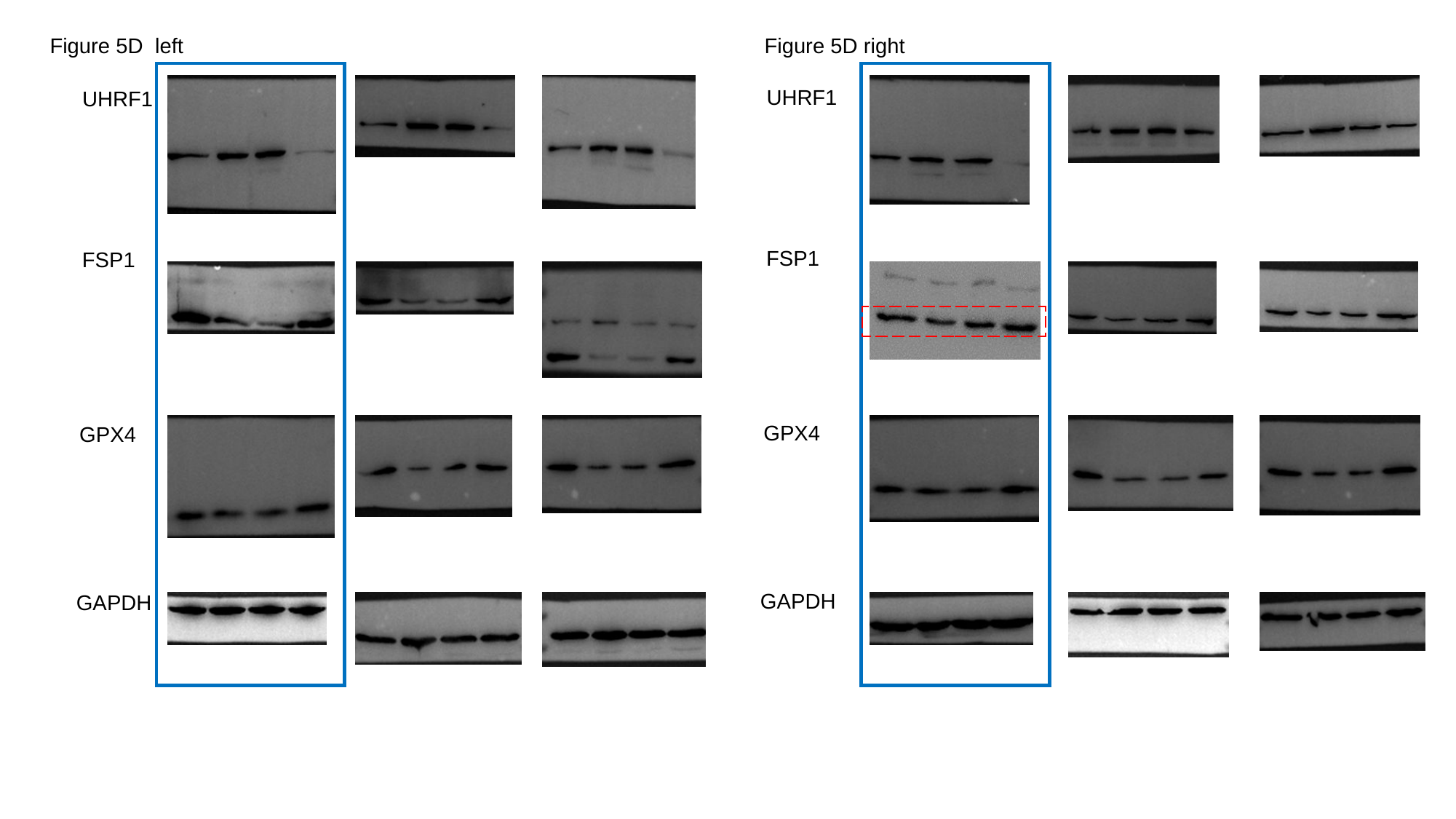

Figure 5D left
Figure 5D right
UHRF1
UHRF1
FSP1
FSP1
 GPX4
 GPX4
GAPDH
GAPDH

## Slide 8
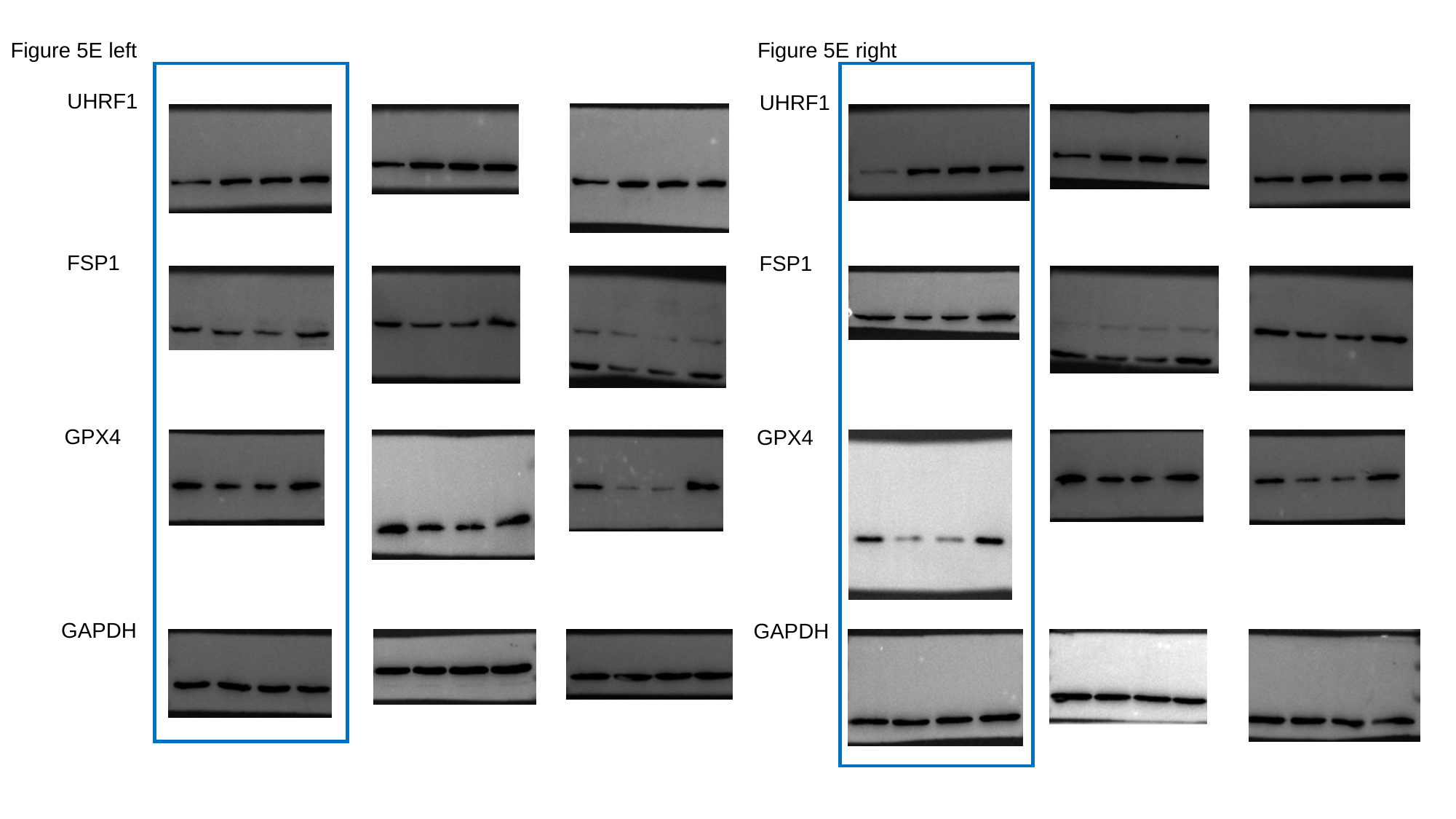

Figure 5E left
Figure 5E right
UHRF1
UHRF1
FSP1
FSP1
 GPX4
 GPX4
GAPDH
GAPDH

## Slide 9
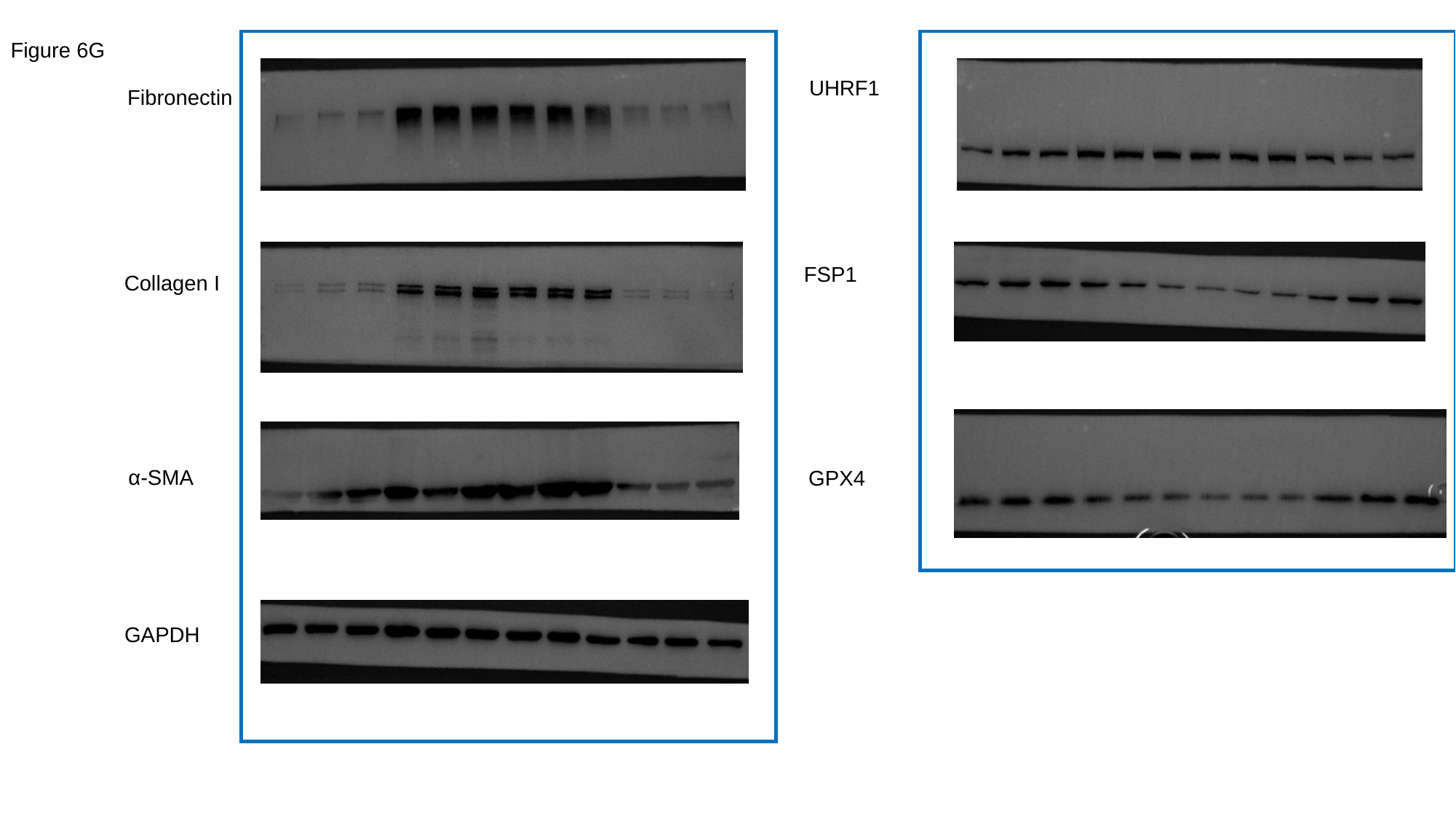

Figure 6G
Fibronectin
UHRF1
Collagen I
FSP1
α-SMA
 GPX4
GAPDH

## Slide 10
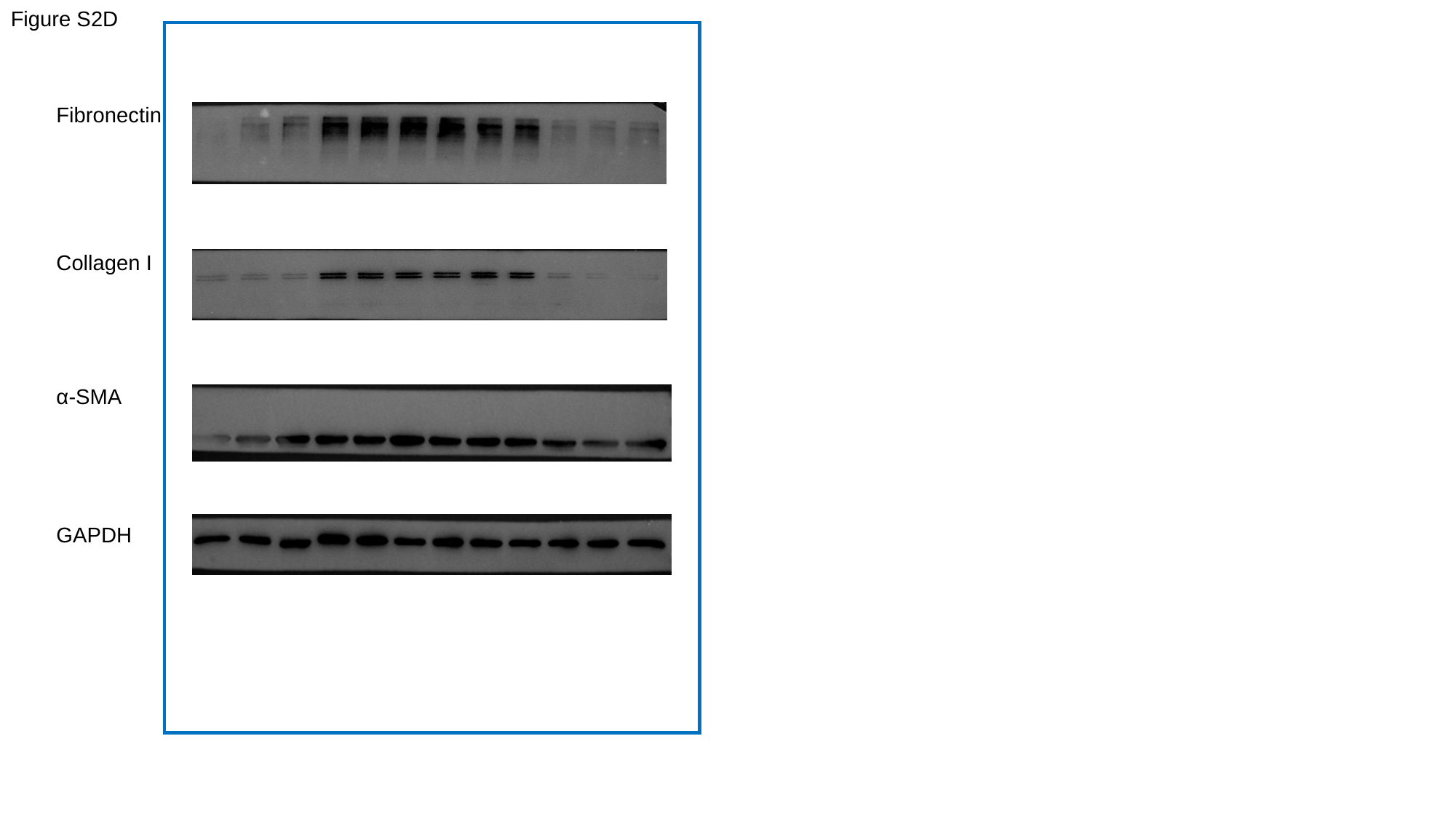

Figure S2D
Fibronectin
Collagen I
α-SMA
GAPDH

## Slide 11
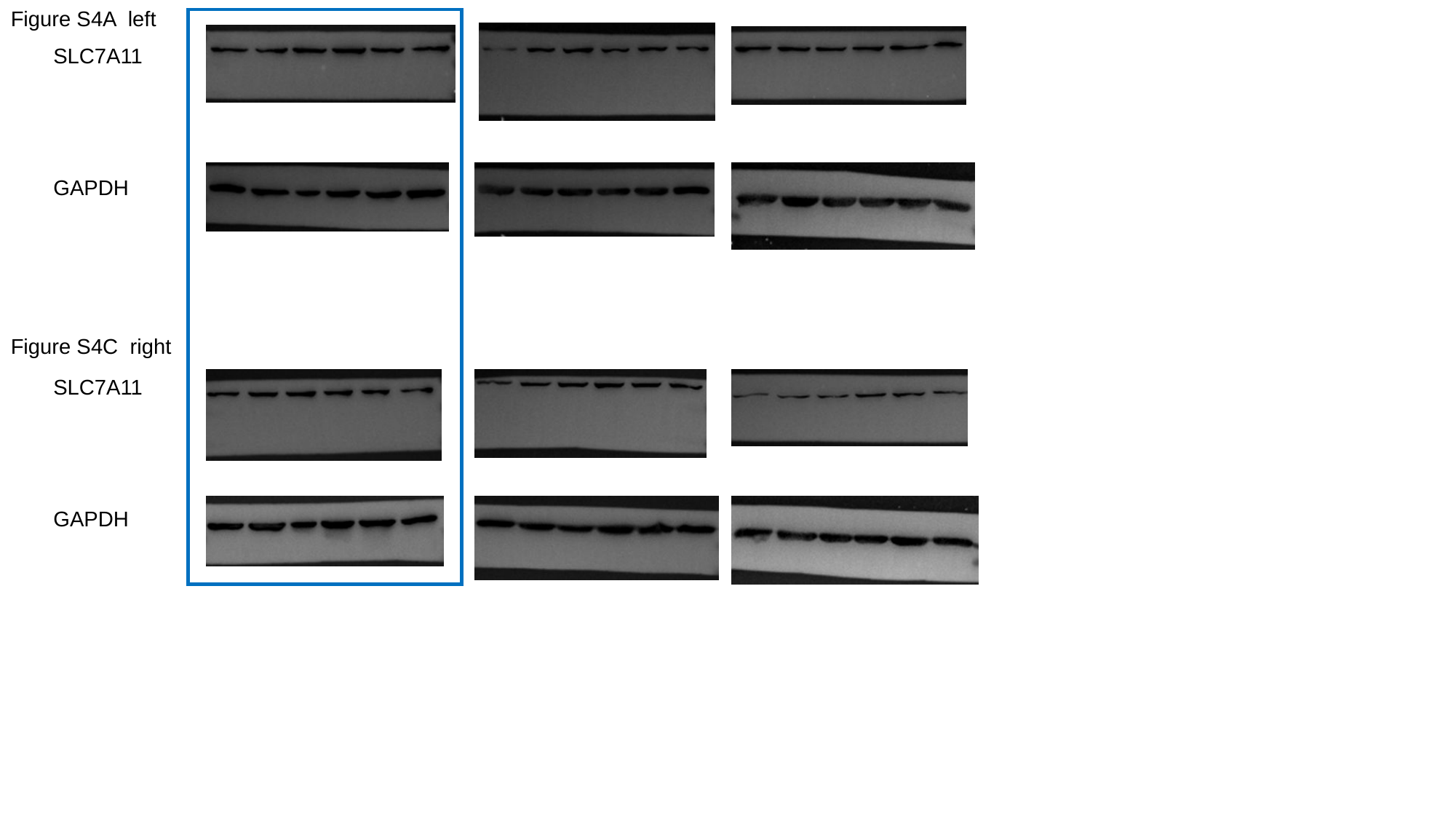

Figure S4A left
SLC7A11
GAPDH
Figure S4C right
SLC7A11
GAPDH

## Slide 12
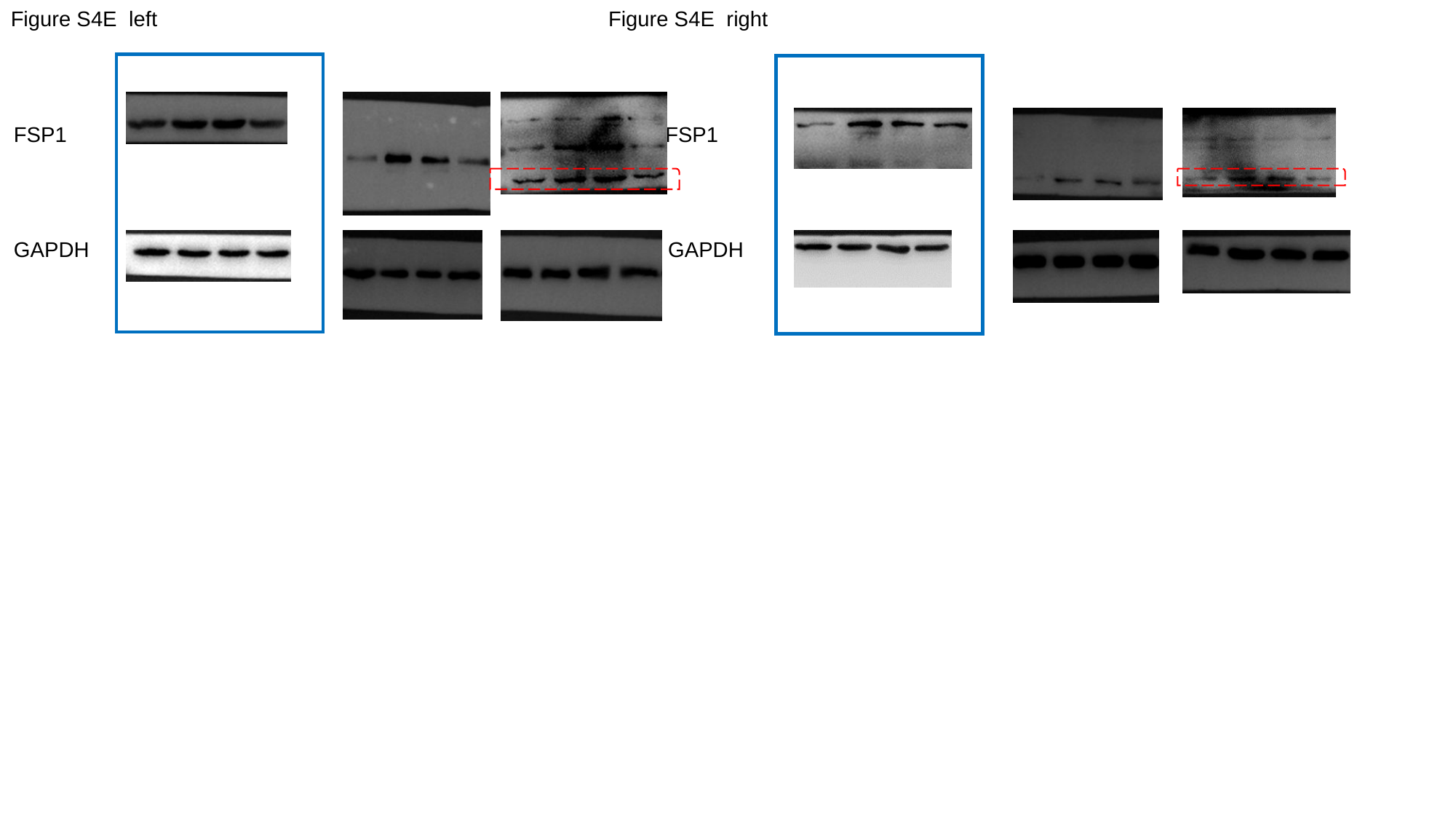

Figure S4E left
Figure S4E right
FSP1
FSP1
GAPDH
GAPDH

## Slide 13
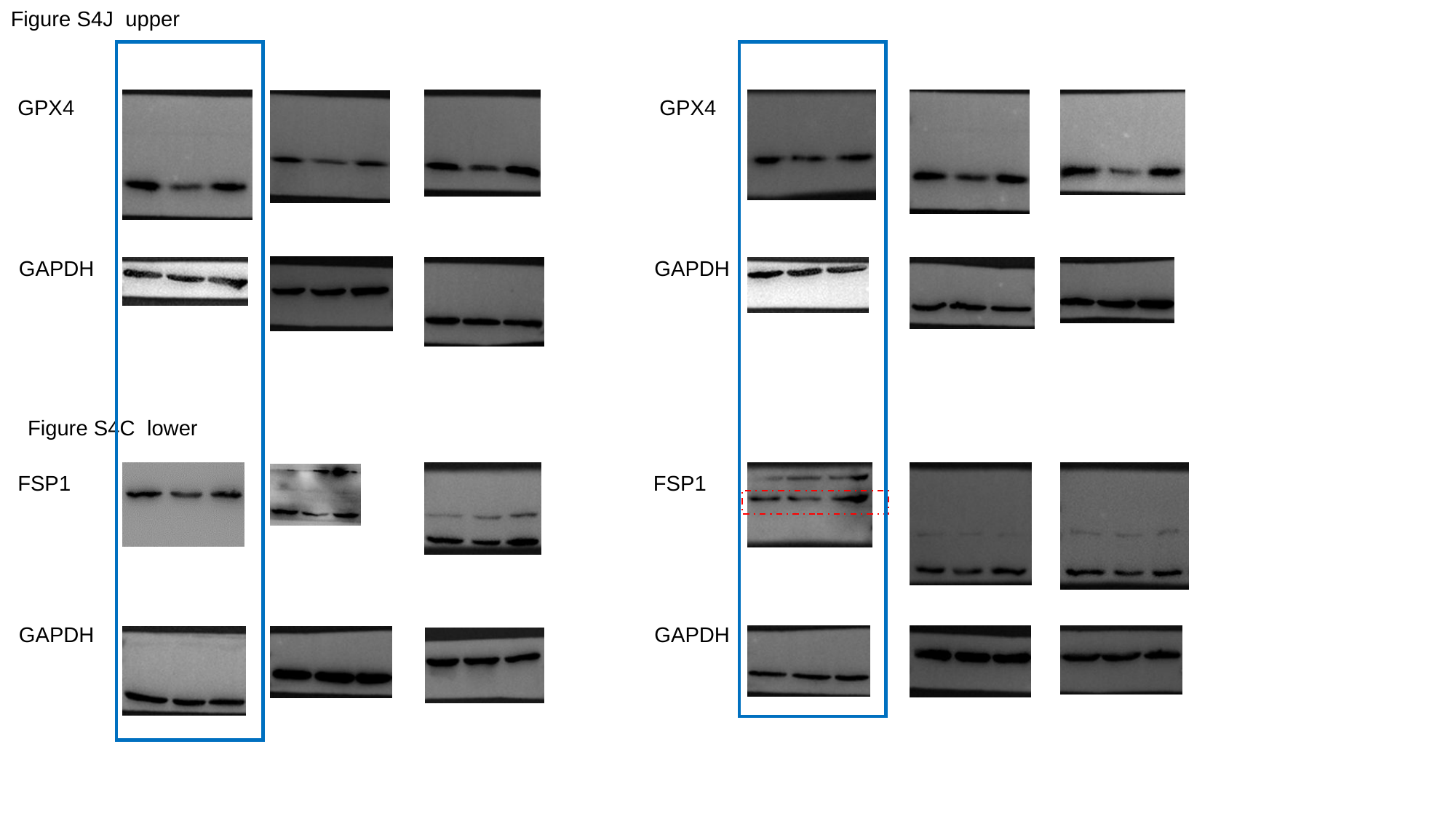

Figure S4J upper
GPX4
 GPX4
GAPDH
GAPDH
Figure S4C lower
FSP1
FSP1
GAPDH
GAPDH

## Slide 14
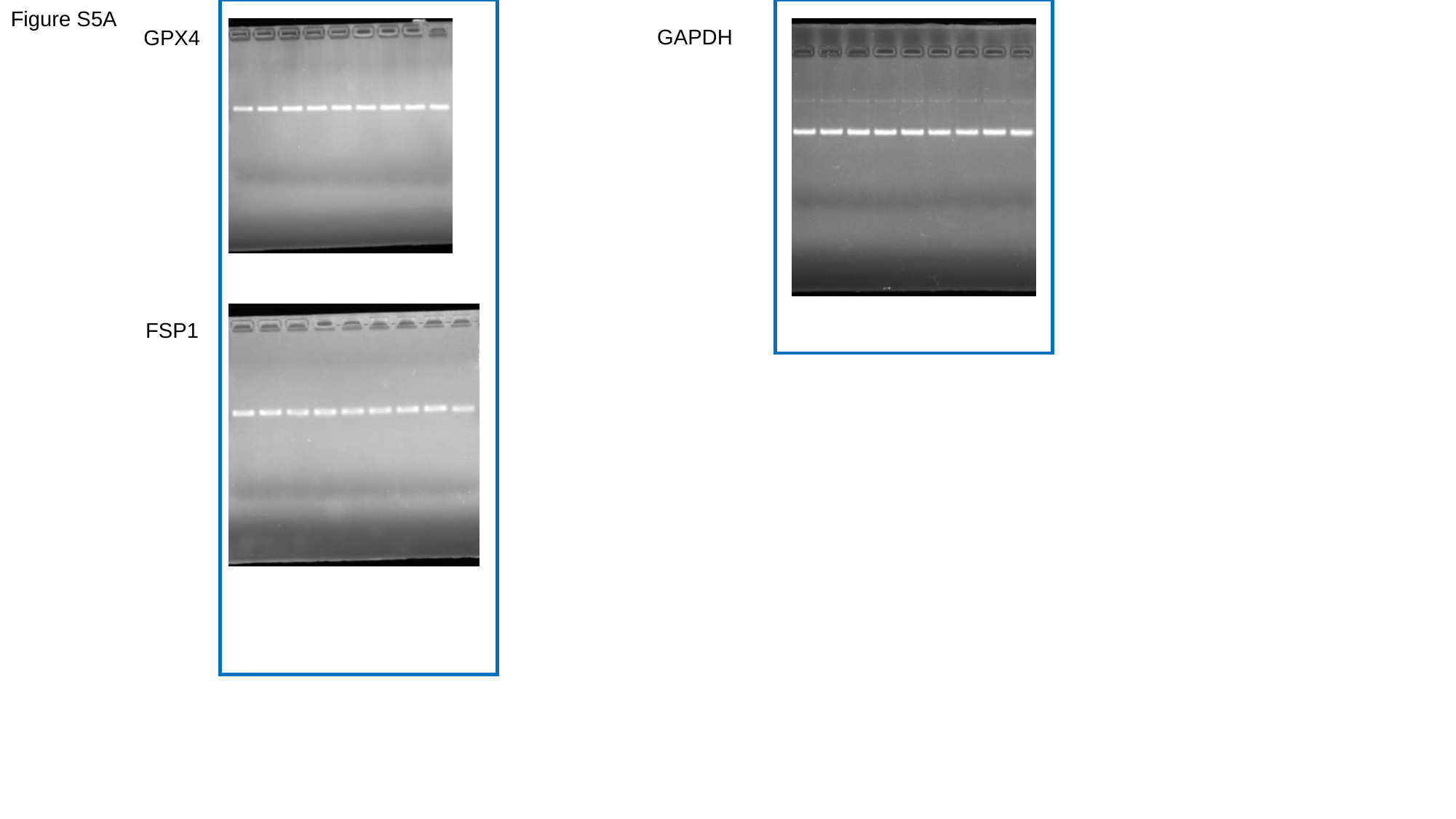

Figure S5A
GAPDH
GPX4
FSP1

## Slide 15
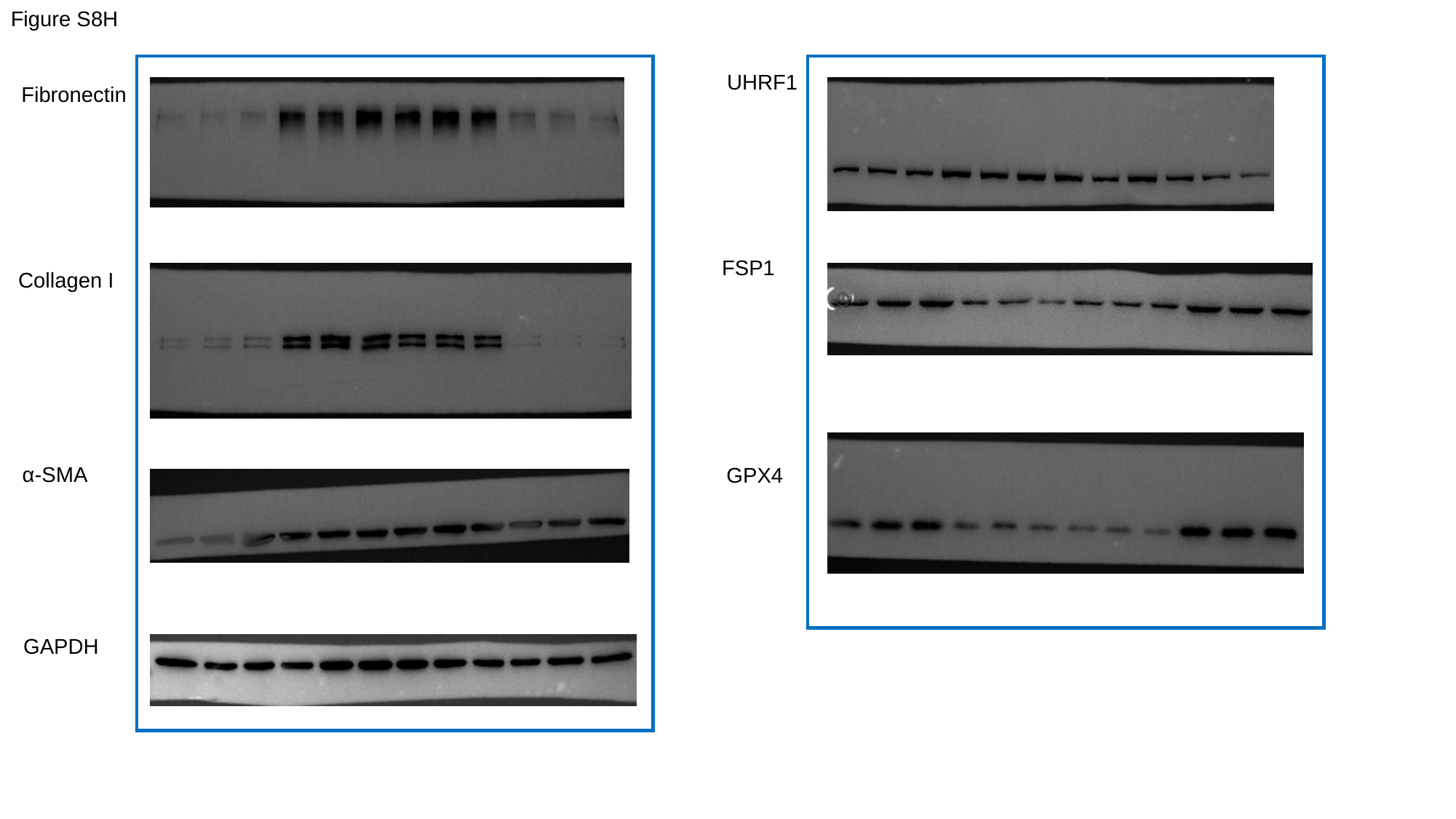

Figure S8H
Fibronectin
UHRF1
Collagen I
FSP1
α-SMA
 GPX4
GAPDH
